# Effects of salinity and broad-range antibiotics on oxalate production, transport, and degradation in *Poecilia latipinna*

**DOI:** 10.1371/journal.pone.0347147

**Published:** 2026-04-17

**Authors:** Felicia Vimala Rajan, Carol Bucking

**Affiliations:** Department of Biology, York University, Toronto, Ontario, Canada; Laurentian University, CANADA

## Abstract

Oxalate is an anion that readily binds calcium and is thought to contribute to osmoregulation. This study investigated how environmental salinity influences oxalate homeostasis in euryhaline sailfin mollies (*Poecilia latipinna*), with a focus on the interplay between microbial symbiosis and host transport processes. Gut microbiome profiling demonstrated regional specialization, with the posterior intestine enriched in oxalate-degrading bacterial families. Community shifts across salinities suggests functional redundancy and resilience, ensuring maintenance of oxalate-catabolizing capacity. Antibiotic treatment disrupted this system, impairing microbial degradation and causing systemic oxalate stress. Oxalate concentrations were also measured in the liver, intestine, and kidney, organs central to oxalate metabolism, under freshwater and seawater conditions. Salinity induced a redistribution of oxalate among these organs, with the gut assuming an auxiliary excretory role in seawater. This functional shift parallels mammalian colon physiology and highlights the gut’s role in balancing ion and oxalate flux. Expression analyses of the oxalate transporters SLC26A3 (solute carrier family 26, member 3) and SLC26A6 (solute carrier family 26, member 6) revealed organ-specific and salinity-dependent regulation. Both transporters displayed distinct responses to seawater exposure, indicating specialized roles in oxalate handling. These patterns suggest coordinated but nonredundant mechanisms that govern absorption and secretion, linking salt transport with oxalate clearance. These findings underscore the microbial contribution to oxalate balance and reveal that osmoregulatory challenges shape gut microbial composition and function. Collectively, this study presents the first comprehensive analysis of oxalate metabolism in a euryhaline teleost and demonstrates a coordinated host–microbe system that mitigates oxalate accumulation across salinities. By integrating metabolic and osmoregulatory demands, *P. latipinna* reallocates excretory function from kidney to gut and leverages microbial symbiosis to preserve homeostasis. These findings expand our understanding of teleost physiology and highlight oxalate metabolism as a critical axis of environmental adaptation.

## Introduction

Oxalate is a metabolite obtained through the diet or through amino acid catabolism in the liver [1,2], that poses unique challenges and opportunities for animals, as excess oxalate can precipitate with calcium to form crystals that can pose health challenges and/or be used for physiological processes. In mammals, the homeostasis of oxalate is tightly regulated through coordinated production, intestinal transport, renal excretion, and microbial degradation [3]. Excess oxalate is primarily excreted via the kidneys, but under conditions of impaired excretion, hyperoxaluria can lead to kidney stone formation and renal damage [4]. Teleost fishes, especially euryhaline species like *Poecilia latipinna* (sailfin molly; [5,6]), offer an alternative model to study oxalate metabolism in the context of osmoregulation. Euryhaline fish regularly transition between freshwater (FW) and seawater (SW) environments, necessitating physiological adjustments to maintain ion and water balance as a consequence of the severe osmotic stress these environments impose. In freshwater, gills express ion uptake transporters [7,8] and animals do not drink water [8,9], while also producing dilute urine [10,11] to fight the constant diffusive loss of ions and gain of water. In seawater, gill transporters facilitate salt excretion [12,13] while animals drink water [9,13] and produce minimal, concentrated urine [10,11] to fight water loss and ion gain. These adjustments could plausibly alter oxalate handling wherein marine teleosts may have a limited renal excretion of anions suggesting that oxalate excretion and metabolism might shift toward extra-renal routes (intestine or bile; [2]).

In mammals, trans-epithelial oxalate transport is primarily mediated by two apical anion exchangers of the SLC26 family: SLC26A6 and SLC26A3 [14]. SLC26A6 is abundantly expressed on the brush border of the small intestine and functions as a Cl^-^/oxalate exchanger, secreting oxalate into the intestinal lumen [15,16,17]. Indeed, mouse models have demonstrated that SLC26A6 is critical for preventing oxalate over-accumulation as knockout animals exhibit excessive intestinal oxalate absorption, leading to elevated plasma and urinary oxalate and calcium-oxalate kidney stones [18,19]. Conversely, SLC26A3, is a Cl^-^/HCO3^-^ exchanger highly expressed in the distal gut that appears to facilitate oxalate absorption. Recent studies show that SLC26A3 knockout mice have reduced intestinal oxalate absorption and a ~ 66% decrease in urinary oxalate excretion relative to wild-type [17]. Together, these findings underscore a dual model in mammals: SLC26A6 serves as a major route for oxalate secretion to limit systemic levels, while SLC26A3 contributes to oxalate absorption [14]. The balance of their activity helps determine whether an animal eliminates oxalate or retains it, and imbalances can lead to hyperoxaluria and nephrolithiasis [14]. This mammalian paradigm provides an intriguing opportunity to examine oxalate homeostasis in fish as these same transporters play a role in environmentally-dependent osmoregulation.

Marine teleosts rely on intestinal Cl ⁻ /HCO₃ ⁻ exchange to absorb water and precipitate excess calcium (and Mg^2+^) as calcium/magnesium carbonate and calcium/magnesium oxalate [2,20,21], a process essential for life in high-salinity water [22,23]. Bicarbonate secretion plays a key role in these processes by increasing intestinal pH (to ~8–9; [24]) which promotes precipitation, lowering luminal osmotic pressures to drive water absorption [25]. This secretion is mediated by SLC26A3 and SLC26A6 [2,21,26,27,28], and while these exchangers have been extensively studied in relation to bicarbonate transport, their role in oxalate homeostasis remains unclear. Notably, their expression is modulated by environmental salinity (e.g., [26,27]) suggesting that if teleost fish also utilize these transporters for oxalate [2], oxalate secretion into the intestine may be salinity-dependent.

Furthermore, the dependence on the intestine for oxalate handling in teleost fish might also be salinity-dependent. Given that freshwater teleosts excrete large volumes of dilute urine (e.g., [11]), they likely eliminate oxalate predominantly via the renal pathway [10,29]. In contrast, seawater-acclimated fish produce minimal, highly concentrated urine (e.g., [30,31,32,33]), limiting their capacity for urinary oxalate excretion and increasing the potential for oxalate accumulation [1,34]. This suggests that seawater teleosts may compensate by enhancing intestinal oxalate secretion [2]. Considering another critical dimension of oxalate homeostasis is microbial degradation in the intestine, this may result in alterations in the gastrointestinal microbiome of fish.

Mammals lack endogenous enzymes to catabolize oxalate, instead relying on oxalate-degrading bacteria in the colon (most famously *Oxalobacter formigenes*) to eliminate oxalate and mitigate absorption [2,35,36,37]. In fact, dysbiosis in the gut microbiome has been associated with calcium oxalate stones [38]. While *O. formigenes* has not been detected in the gut microbiome of *P. latipinna* [2] [2], other bacterial taxa, including members of the *Lactobacillus* genus and certain *Desulfovibrio* strains, exhibit oxalate-degrading capabilities [39,40,41,42]. Importantly, past piscine studies suggest that microbial community composition shifts in response to salinity [43,44,45,46,47,48], potentially altering the capacity for oxalate degradation under different osmoregulatory conditions. Indeed, specifically members of the *Lactobacillus* genera (including bacteria such as *L. acidophilus* and *L. casei* that have oxalate-degrading capabilities; 40.) have been shown to increase with increasing salinities [41,42].

Finally, salinity may also alter the amount of oxalate consumed and/or produced by fish. Feeding rates are known to be influenced by salinity [49,50,51,52] and for an herbivorous fish that consumes plant materials known to be high in oxalate (like *P. latipinna*), this may increase the dietary burden of oxalate in the intestine. Furthermore, oxalate is a metabolic by-product of amino acid catabolism in the liver [1,2]. Specifically, the enzyme lactate dehydrogenase (LDH) catalyzes the conversion of glyoxylate, an intermediate in amino acid metabolism, into oxalate [4,53]. During seawater acclimation, liver LDH activity is known to increase in euryhaline fish like the rainbow trout [54,55].

As a result, we hypothesize that acclimation to higher salinity will trigger physiological changes (in both transporters and gut microbiota) that favor intestinal oxalate excretion. We predicted that the gut microbiome of seawater fish would harbor a greater abundance of oxalate-degrading bacteria to aid in enhanced intestinal handling of oxalate, and we further hypothesized that disruption of the microbiome would impair oxalate degradation in the gut, leading to increased oxalate accumulation in the fish. During the transition to seawater acclimation, *P. latipinna* were predicted to exhibit higher oxalate concentrations in blood plasma, liver, intestines, and kidneys due to impaired urinary excretion. Once acclimated to seawater, fish were predicted to upregulate intestinal SLC26A6 expression, enhancing oxalate secretion, while downregulating SLC26A3 to limit intestinal oxalate absorption. This study addresses major knowledge gaps through novel examination of oxalate production, transport, and degradation in *P. latipinna* under freshwater versus seawater conditions, and with or without antibiotic-mediated microbiome ablation. The findings provide novel insight into teleost osmoregulatory physiology, highlighting oxalate as a previously underappreciated metabolite at the nexus of ion regulation and microbial symbiosis.

## Materials and methods

All experiments and animal care were conducted in accordance with York University’s institutional guidelines and regulations under an approved Animal Use Protocol (Research Ethics Approval Number: 2017−14 (R1)). This study is reported in accordance with the ARRIVE (Animal Research: Reporting of In Vivo Experiments) guidelines.

### Obtaining experimental animals, housing, and fish-care

Freshwater-acclimated sailfin mollies (*Poecilia latipinna*) were obtained from the Fish & Bird Emporium (Churchill, Ontario) and transported to York University (Toronto, Ontario). Upon arrival, these adult (age: ~ 1 year old; weight: ~ 2 g) sailfin mollies were separated by sex and housed in recirculating 10-gallon (37.85 L) glass aquaria (Aqueon; Franklin, Wisconsin) at a stocking density of 15–20 fish per tank. All tanks contained a sponge filter with aeration and a water heater set to 24°C.

Fish were acclimated to dechlorinated Toronto municipal freshwater for at least two weeks before experimentation. Water quality parameters (ammonia, nitrite, and nitrate levels) were monitored using the Freshwater Aquarium Master Test Kit and Saltwater Aquarium Master Test Kit (API®; Chalfont, Pennsylvania) for freshwater and seawater conditions, respectively. Water changes were performed daily or every other day during the first 2–3 days after arrival to minimize ammonia buildup. Once ammonia levels stabilized, 50% of the water was replaced weekly, and filters were cleaned as needed.

Fish were fed once daily at 12:00 PM with 1 mm pellets of *Premium Fish Food – Veggie Formula* (NorthFin™; Toronto, Ontario) at a ration of 5% body weight to ensure satiation. A 14:10 light:dark cycle was maintained to promote normal circadian rhythms.

### Acclimation of animals to control and treatment conditions

Following the 2-week lab-acclimation period, the animals were randomly assigned to 1 of 4 treatments: freshwater for another 14 days (FW14; control), seawater acclimation for 14 days (SW14), seawater acclimation for 28 days (SW28), and seawater acclimation for 14 days with broad-range antibiotic treatment (SW14 + Antibiotics). For each treatment group, fish were housed in tanks separated by sex, with three fish per tank (S1 Table in Supplemental Information). For the fish utilized in measuring oxalate concentrations, each experimental condition was represented by ten replicate tanks (5 male tanks and 5 female tanks). For the gut microbiome analyses, each experimental condition was represented by five replicate tanks (3 male tanks and 2 female tanks). For the qPCR, each experimental condition was represented by 6 tanks (3 male tanks and 3 female tanks). Fish were randomly selected from these tanks for sampling, with equal numbers of males and females used for qPCR and measuring oxalate concentrations. Since fish were sampled from sex-segregated tanks, statistical analyses were conducted using hierarchical models that accounted for within-tank correlation by including tank identity as a random effect in linear mixed-effects models where assumptions were satisfied and generalized additive mixed models when assumptions of linearity were not met, following established analytical approaches in fish physiology studies [56,57]. No significant sex effects or tank effects were observed for the measured endpoints.

Seawater acclimation was achieved by gradually increasing salinity to 35 ppt over two days. Hypersaline (~85 ppt) water was prepared using 0.2 µm filtered dechlorinated water and Instant Ocean Sea Salt Mix (Instant Ocean; Blacksburg, Virginia). Approximately 25% of the tank water was replaced with hypersaline water at 12-hour intervals, increasing salinity by ~18 ppt per day. Salinity was measured using a handheld refractometer (Fisher Scientific, Pittsburgh, Pennsylvania).

Antibiotics were administered directly into the tank water at low mg/L concentrations, an approach consistent with prior aquaculture and fish microbiome research using antibiotic exposures in fish systems [58]. For the antibiotic treatment, a broad-spectrum antibiotic mixture was added to the tank water to suppress gut bacterial populations. The antibiotic solution was prepared by dissolving 0.1 g ampicillin (Millipore Corp., St. Louis, Missouri), 0.1 g gentamycin (Fisher BioReagents, Pittsburgh, Pennsylvania), 10 mL penicillin-streptomycin solution (HyClone, Marlborough, Massachusetts), and 0.02 g kanamycin (Thermo Scientific, Whitby, Ontario) in 100 mL of tank water. This antibiotic solution was added daily to 37.85 L (10-gallon) fish tanks at feeding time, resulting in final in-tank concentrations of approximately 2.64 mg/L ampicillin, 2.64 mg/L gentamycin, 2,644 U/L penicillin, 2.64 mg/L streptomycin, and 0.53 mg/L kanamycin. Fish food pellets were soaked in a small aliquot (15mL) of this antibiotic solution for 3 minutes prior to feeding. Antibiotic exposure continued once daily for 14 consecutive days.

### Dissections

Fish were randomly selected for sampling across all conditions. Euthanasia was performed prior to feeding (~11:00 AM) using a buffered high-dose treatment (2.0 g/L) of tricaine methanesulfonate (MS-222; Syndel Canada, Nanaimo, British Columbia) dissolved in either freshwater or seawater, depending on the fish’s acclimation condition. The pH of the anesthetic solution was adjusted to ~7.5 for freshwater fish and ~8.5 for seawater fish using NaOH (Fisher Scientific, Pittsburgh, Pennsylvania). Following euthanasia, a spinal cord transection was performed.

All dissections were conducted using sterile techniques. Dissection instruments (forceps, scissors, tweezers) were soaked in 70% ethanol for 15 minutes, then UV-sterilized under a fume hood (UV-PCR Workstation; Fisher Scientific, Whitby, Ontario) for 20 minutes before use. Instruments were also decontaminated in 70% ethanol between each dissection.

Urine was collected immediately following euthanasia by carefully cannulating the urogenital pore with a sterile Western blot pipette tip (FisherBrand, Fisher Scientific, Whitby, Ontario) attached to a 1 mL syringe (Henke Sass Wolf, Tuttlingen, Germany). Due to the small size of the fish, urine volumes were limited (~10–30 µL per fish); all obtainable urine was collected from each individual. Samples were immediately transferred to sterile microcentrifuge tubes and stored at −20°C until analyzed using the *Oxalate Assay Kit*. To minimize contamination, collection tools were changed between fish, and care was taken to avoid contact with surrounding tissues. Blood was collected from fish acclimated to each of the four conditions for subsequent use in the *Oxalate Assay Kit*. The blood was collected from the initial spinal cut at the base of the head using a sterile 1mL Insulin Safety Syringe from Covidien (Dublin, Ireland). The blood samples were centrifuged using a benchtop centrifuge (Fisherbrand™ Mini-Centrifuge 100-240V, 50/60 Hz, Mississauga, Ontario) for ~1 minute to separate the plasma from the red blood cells. The plasma samples were stored at −20ºC until they were used in the *Oxalate Assay Kit.* During dissection, the anterior intestines, posterior intestines, whole kidneys, and livers were harvested, flash-frozen using dry ice and then stored at −80ºC until the tissues were either used in the *Oxalate Assay Kit* (Sigma-Aldrich, Burlington, Massachusetts) or with Trizol (Invitrogen; Waltham, Massachusetts) for RNA-extractions. Intestinal tissues from the FW14 and SW14 conditions were also flash-frozen using dry ice and then stored at −80ºC until the gDNA was extracted for 16S metagenomic sequencing.

To assess the impact of broad-range antibiotic exposure on intestinal bacterial presence, PCR targeting the bacterial 16S rRNA gene was performed on intestinal gDNA from four sentinel fish (one male and one female from each of the SW14 and SW14 + Antibiotics conditions) and visualized by agarose gel electrophoresis. This approach served as a qualitative presence/absence screen to confirm that antibiotic treatment had a detectable effect on intestinal bacterial DNA and was not intended as a quantitative measure of bacterial load, consistent with prior studies demonstrating that 16S PCR can reliably detect reductions in bacterial DNA following antibiotic treatment [59,60].

### Genomic DNA (gDNA) extractions for bacterial presence/absence

Genomic DNA (gDNA) was extracted from intestinal tissues to assess bacterial presence and for downstream 16S metagenomic sequencing and QIIME2 (Quantitative Insights Into Microbial Ecology 2) analyses. Intestinal samples from the FW14 (for gut microbiome analyses), SW14 (for gut microbiome analyses), and SW14 + Antibiotics conditions were processed using Qiagen’s Soil Kit Pro (Qiagen; Germantown, Maryland) according to the manufacturer’s protocol, with sterile techniques maintained throughout under a UV fume hood. Instruments (forceps, tweezers, scissors) were decontaminated by soaking in 70% ethanol for 15 minutes and UV sterilized for 20 minutes prior to use.

Small pieces of the intestinal sample were placed into a PowerBead Pro Tube containing solution CD1, vortexed to disperse the tissue and solubilize contaminants (e.g., humic acids), and then homogenized horizontally at maximum speed (Fisher Vortex 12–812 – Genie 2; Fisher Scientific) for 20 minutes. The homogenate was centrifuged, and the supernatant was mixed with solution CD2, which precipitates inhibitors such as humic substances, followed by another centrifugation step. After adding solution CD3 (a high-salt solution) to the cleared supernatant, the mixture was loaded onto an MB Spin Column. Under high-salt conditions, DNA binds to the silica membrane while contaminants pass through during centrifugation. The column was subsequently washed with solution EA (to remove proteins and contaminants) and solution C5 (an ethanol-based wash) before a final centrifugation to eliminate residual ethanol. DNA was then eluted using solution C6 (10 mM Tris, no salt) and stored at −20°C until further use.

Blank extractions (without tissue) were performed alongside each batch to monitor for contamination. All centrifugation steps were conducted at room temperature using an Eppendorf Centrifuge 5415D (Eppendorf Canada, Mississauga, Ontario).

### Polymerase chain reaction (PCR) and gel electrophoresis

To confirm bacterial presence or absence, gDNA extracted from SW14 and SW14 + Antibiotics samples was subjected to PCR using universal bacterial primers (8F and 533R; Sigma-Aldrich, St. Louis, Missouri) targeting the 16S rRNA gene (S2 Table in Supplemental Information). PCR was performed with DreamTaq Green PCR Master Mix (Thermo Fisher Scientific, Whitby, Ontario) following the manufacturer’s protocol, with an annealing temperature of 55.5°C for 25 cycles. Reactions were run on an Eppendorf Mastercycler® gradient thermal cycler (Eppendorf Canada Ltd., Mississauga, Ontario).

PCR products were resolved on a 1.5% agarose gel stained with ethidium bromide (50 µg/100 mL gel; Thermo Fisher Scientific) in 1X Tris-acetate-EDTA (TAE) buffer at 70V for 30 minutes. A 100 bp DNA Ladder (O’RangeRuler; Thermo Fisher Scientific) was run alongside the samples. Gels were imaged under UV light using the MiniBIS Pro (DNR Bio-Imaging Systems, Neve Yamin, Israel). The presence of a band indicated detectable bacterial DNA, while its absence in the SW14 + Antibiotics group confirmed the effectiveness of the treatment.

The same extraction protocol was used for samples from the anterior and posterior intestines of FW14 and SW14 fish (n = 5 per intestinal section per condition, 20 samples total) to verify successful bacterial DNA recovery prior to submission for 16S metagenomic sequencing at Genome Quebec.

### QIIME2 and PICRUSt2 Analyses

Raw 16S rRNA gene sequencing reads have been deposited in the NCBI Sequence Read Archive under BioProject accession number PRJNA1406477.

Microbiome bioinformatic analyses were conducted using the QIIME2 (version 2023.5) pipeline [61]. Raw sequence data was obtained from 16S metagenomic sequencing (NextSeq PE300 bp 5 million reads total for 20 samples; ~ 250,000 reads/sample; Genome Quebec, Quebec, Canada). There were a total of 5,623,878 reads and 4,914,547 passed quality filtering, resulting in ~87% of reads being used in subsequent analyses. Further, ~ 77% of the sequences were non-chimeric and were demultiplexed using the q2‐demux plugin followed by denoising the sequences with DADA2 using the q2-dada2 plugin [62]. Amplicon sequence variants (ASVs) were aligned with MAFFT [63] and a phylogeny constructed using FastTree2 [64].

Alpha diversity (e.g., Shannon Diversity Index) and beta diversity metrics (e.g., Bray–Curtis Dissimilarity) were computed using QIIME2’s diversity plugin. Samples were rarefied to 131,529 sequences per sample before Principle Coordinate Analysis (PCoA). Taxonomy was assigned to ASVs with the q2-feature-classifier using a naïve Bayes classifier trained on the SILVA132 database [65].

ANCOM (Analysis of Composition of Microbiomes [66]) was employed to determine significant differences between the anterior and posterior intestinal sections of fish acclimated to FW14 versus SW14. Functional predictions of the gut microbiome were further explored using PICRUSt2 (Phylogenetic Investigation of Communities by Reconstruction of Unobserved States 2) [67] to examine pathways related to metabolism, digestive system, transport and catabolism, protein digestion, amino acid metabolism, and glyoxylate and dicarboxylate metabolism. Graphical representations of these functional changes were generated using STAMP (Statistical Analysis of Metagenomic Profiles [68,69]).

### Oxalate assays

Oxalate concentrations were determined using the *Oxalate Assay Kit* (Sigma-Aldrich, Burlington, Massachusetts). Free (unprecipitated) oxalate concentrations were measured in the anterior intestine, posterior intestine, kidney, and plasma. Whole tissues were homogenized in sterile water using a glass homogenizer, and plasma samples were diluted accordingly. All dilutions and tissue weights were accounted for in the oxalate concentration calculations. Oxalate concentrations were measured in micromolar (µM) using the *Oxalate Assay Kit* and normalized to the wet weight of the tissue (expressed as µmol mg wet tissue weight ⁻ ¹). This approach has precedent in the literature (mongrel dogs [70] and Sprague-Dawley rats [71]), where similar normalization was applied to express oxalate concentrations relative to tissue mass.

Total oxalate content was determined by dissolving precipitated oxalate using hydrochloric acid (HCl). Since calcium oxalate is soluble at pH ≤ 3 [72], the tissue homogenate pH was gradually lowered to ~2.4–2.5 using 10 mM HCl. The oxalate concentration plateaued at this pH, confirming complete dissolution.

The precipitated oxalate concentration was calculated as the difference between total and free oxalate for each tissue type. Oxalate concentrations were quantified using spectrophotometry (Synergy HT Multi-Mode Microplate Reader; BioTek, Winooski, Vermont) at 595 nm, per the kit manufacturer’s protocol.

### Primer design

De novo primers were designed for SLC26A3 and SLC26A6 using Ensembl and NCBI databases. Primers for SLC26A6 and SLC26A3 were designed in accordance with standard quantitative PCR criteria, including appropriate melting temperature, GC content, amplicon size, and avoidance of secondary structures. To prevent amplification of genomic DNA, the forward and reverse primers were placed in separate exons with an intron located between them. Due to the high sequence conservation among isoforms of both genes, it was not possible to design isoform-specific primers that satisfied these requirements (S1 Fig). Therefore, a single primer set was designed within a conserved region shared across all isoforms of each gene, enabling reliable amplification and quantification of the total transcript pool (S1 Fig).

Regular PCR validation (using Using DreamTaq Green PCR MasterMix (2X; Thermofisher Scientific, Whitby, Ontario)), confirmed single-product amplification, and PCR products were purified (GeneJET PCR Purification Kit; Thermo Fisher Scientific, Whitby, Ontario) and sequenced at the TCAG-DNA Sequencing Facility (Toronto, Ontario). The primer sequences of SLC26A6 (product length: 233 bp) and SLC26A3 (product length: 216 bp) are in S2 Table (Supplemental Information).

All other primers used in this study were previously designed, sequenced, and validated in our lab (Bucking Lab at York University, Toronto, Ontario), including the universal bacterial primers, 8F and 533R for the 16S rRNA gene as well as the primers for the 18S, RPL7 and RPL17 genes in the sailfin molly.

### RNA extractions, cDNA synthesis, and quantitative PCR (qPCR)

RNA was extracted from intestinal and kidney tissues using TRIzol (Invitrogen, Waltham, Massachusetts) and quantified spectrophotometrically. Further, using the absorbance values ratios (260nm: 280nm), and visual determination using an agarose gel, RNA quality was subsequently determined. After the RNA quality was checked and the concentration (ng/µL) determined, cDNA was synthesized using half reactions of Promega’s *cDNA Synthesis Kit*.

The three reference genes that were used for qPCR are 18S (ribosomal RNA gene), RPL7 (ribosomal protein L7 gene), and RPL17 (ribosomal protein L17 gene; S2 Table in Supplemental Information). The 18S, RPL7, and RPL17 primers were previously sequenced and validated in our lab for this species. The optimal annealing temperature for the three reference genes was 60ºC. Primers for the test genes, SLC26A6 (solute carrier family 26, member 6) and SLC26A3 (solute carrier family 26, member 3) were designed and subsequently validated through sequencing. The optimal annealing temperature for both the test genes, SLC26A6 and SLC26A3 was 61ºC. PowerTrack SYBR Green Master Mix (Applied Biosystems, Thermofisher Scientific, Whitby, Ontario) was used for quantitative PCR per the manufacturer’s protocol. All qPCR experiments were run for 40 cycles on the LightCycler® 96 Instrument (Roche; Mississauga, Ontario). Using the LightCycler® 96 SW 1.1 software, melt-curve analysis was conducted to ensure that there was only one product amplified in each qPCR reaction. For both SLC26A3 and SLC26A6 primer sets, negative controls with sterile water were run as well as no reverse transcriptase (NRT) controls using RNA as the template to ensure there was no DNA contamination in the RNA preparation.

To verify that pipetting was precise, the Cq values of each sample’s technical replicates were compared to ensure they were no more than 0.5 Cq apart [73]. The Cq values for each sample’s technical duplicates were then averaged for subsequent use in calculating relative gene expression. Five concentrations (ng/µL) of pooled sailfin molly cDNA (from a 10-fold serial dilution) were used in the efficiency curves for both SLC26A6 and SLC26A3. Standard curves were generated using the LightCycler® 96 SW 1.1 software to determine if primer efficiencies were similar (acceptable range: 90%−110% [74,75]) between the gene of interest (SLC26A3 or SLC26A6) and the housekeeping genes (18S, RPL7, RPL17). The relative gene expression ratios were calculated using the Livak method.

### Statistical analyses and graphing

All oxalate assay, qPCR, and microbiome data were imported into R (version 4.4.1) for statistical analysis. Data transformations (log₁₀ or square root) were applied as necessary to meet normality and other model assumptions. Potential outliers were evaluated using Grubb’s test (GraphPad Prism version 9.3, GraphPad Software, San Diego, California). No significant outliers were identified, and all data points were retained for statistical analyses.

For the qPCR and oxalate concentrations, individual fish were treated as the unit of observation while accounting for potential tank-level clustering by including tank identity as a random effect in linear mixed-effects models; when assumptions of linearity were not met, generalized additive mixed models with random tank effects were used. Fixed effects included treatment, sex, and their interaction, allowing inference at the fish level while appropriately estimating uncertainty associated with the number of tanks, consistent with prior fish physiology studies [56,57]. These models indicated no significant tank effects or sex effects for any dataset, so fish were treated as independent observations for subsequent analyses.

The statistical test utilized for each dataset is indicated within each respective figure legend. Statistical significance was accepted at p < 0.05. Graphs (except Fig 4) were generated in R, with error bars representing the mean ± SEM. For the alpha diversity, the Kruskal-Wallis test was used on the Shannon Diversity Index to compare the conditions. The beta-diversity was assessed using Bray-Curtis dissimilarity and visualized with principal coordinate analysis (PCoA). PICRUSt2 data (for the digestive system, transport and catabolism, protein digestion and absorption, amino acid metabolism, and glyoxylate and dicarboxylate metabolism) were analyzed in STAMP using two-way ANOVAs (Analysis of Variance) with Tukey post-hoc tests [71,76], with corresponding graphs (Fig 4) produced in STAMP. Sample sizes (n-values) are reported in each figure legend.

The PICRUSt2 data was also imported into R and the top 15 (based on highest mean proportions) predicted differential KEGG (Kyoto Encyclopedia of Genes and Genomes) pathways (Level 2 based on the full set of abundance data) between the freshwater and seawater environments for the anterior and posterior intestines; as well as intestinal zonation between the anterior and posterior intestines in freshwater and seawater was determined. All predicted differential KEGG pathways (Level 2) under the metabolism category between the freshwater and seawater environments for the anterior and posterior intestines; as well as intestinal zonation between the anterior and posterior intestines in the freshwater and seawater conditions were also identified. The top 15 (based on highest mean proportions) predicted differential KEGG pathways (Level 3) under the metabolism category between the freshwater and seawater environments for the anterior and posterior intestines, as well as intestinal zonation between the anterior and posterior intestines in the freshwater and seawater conditions were compared. A one-way ANOVA with Tukey post-hoc was used for comparisons across environmental conditions (i.e., FW14-anterior vs. SW14-anterior and FW14-posterior vs. SW14-posterior). A two-way ANOVA with Tukey post-hoc was used for comparisons between the anterior and posterior intestine in either freshwater or seawater to reveal patterns of intestinal zonation.

LEfSe (linear discriminant analysis effect size) analyses were conducted on microbiome data using the lefser package in R [77,78]. LDA (linear discriminant analysis) scores were calculated and graphed using ggplot2 in R to determine the significantly different bacterial families between the freshwater and seawater conditions for each of the intestinal sections (anterior and posterior). The ggtree package was used to create phylogenetic cladograms highlighting the significantly different bacterial families between the freshwater and seawater conditions for each of the intestinal sections (anterior and posterior). Further, to examine patterns of zonation along the intestine, LEfSe was also used to determine the significantly different bacterial families between the anterior and posterior intestines of each condition (freshwater and seawater), and the subsequent phylogenetic cladograms were created.

## Results

### Gut microbiome analyses

**Fig 1:**
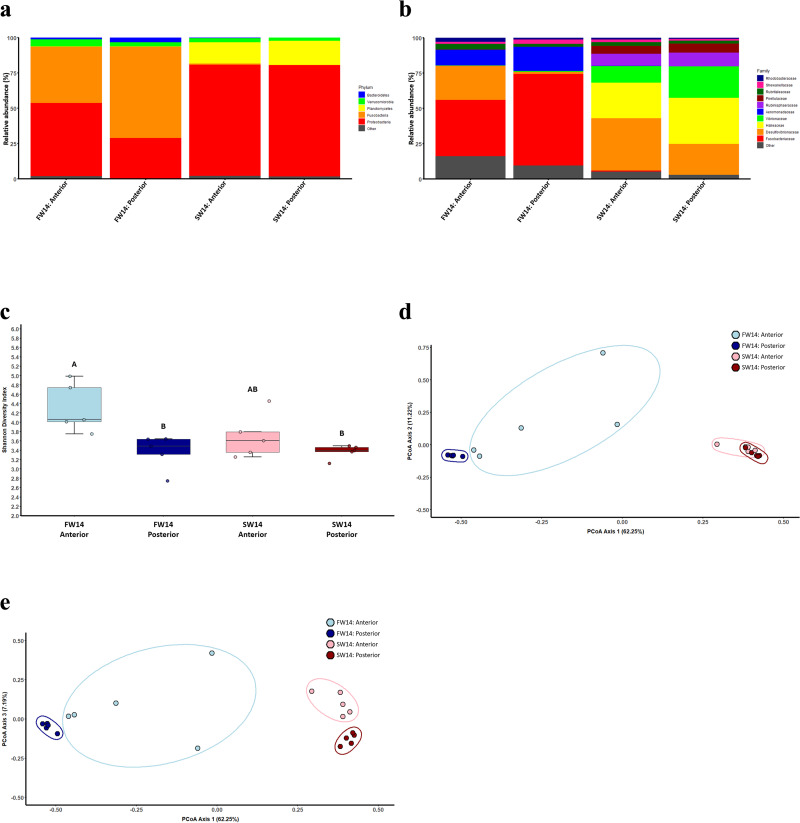
Analyses of the gut microbiome of sailfin mollies acclimated to either freshwater or seawater for 14 days. Taxonomic bar plots displaying the relative abundance (%) of the top five most abundant bacterial phyla (a) and the top ten most abundant bacterial families (b) across the four conditions of: FW14-Anterior, FW14-Posterior, SW14-Anterior, and SW14-Posterior are shown. Alpha-diversity (Shannon Diversity Index; c) was compared across conditions using a Kruskal-Wallis test and conditions that share letters are not significantly different. Beta-diversity was assessed using Bray-Curtis dissimilarity and visualized with principal coordinate analysis (Axis 1 vs. Axis 2; d and Axis 1 vs. Axis 3; e) were also observed across the four conditions.

Taxonomic bar plots show the relative abundance of the top five bacterial phyla (*Bacteroidetes*, *Verrucomicrobia*, *Planctomycetes*, *Fusobacteria*, and *Proteobacteria*; Fig 1a) and top ten bacterial families (*Rhodobacteraceae*, *Shewanellaceae*, *Rubritaleaceae*, *Pirellulaceae*, *Rubinisphaeraceae*, *Aeromonadaceae*, *Vibrionaceae*, *Halieaceae*, *Desulfovibrionaceae*, and *Fusobacteriaceae*; Fig 1b). Alpha-diversity is displayed in the Shannon Diversity Index and there is a clear pattern of zonation in the freshwater condition, but not in the seawater condition (Fig 1c) with diversity in the anterior intestine decreasing with seawater acclimation. Beta-diversity is shown in the Bray-Curtis PCoA and the freshwater intestines (both anterior and posterior) are clearly clustered away from the seawater intestines, indicating differences in the gut microbiomes of these two different environments (Fig 1d).

ANCOM at the family level of bacteria (level 5) was conducted to determine significant changes in the anterior versus posterior sections of sailfin mollies acclimated to either freshwater or seawater for 14 days (S4 Fig). According to the ANCOM at level 5, the following families of bacteria changed in abundance across both the environmental conditions (FW vs. SW) and intestinal sections (anterior vs. posterior intestine): *Aeromonadaceae*, *Akkermansiaceae*, *Bacteroidaceae*, *Barnesiellaceae*, *Burkholderiaceae*, *Chitinbacteraceae*, *Chromobacteriaceae*, *Enterobacteriaceae*, *Fusobacteriaceae*, *Gimesiaceae*, *Halieaceae*, *Microtrichaceae*, *Pirellulaceae*, *Rhizobiales incertae sedis*, *Rikenellaceae*, *Rubinisphaeraceae*, *Stappiaceae*, *Vibrionaceae*, and *Xanthomonadaceae* (S4 Fig). The abundance of *Vibrionaceae* was only 0.38% in the anterior intestine of the FW14 condition but was much higher at 11.29% in the anterior intestine of the SW14 condition (S4 Fig). *Vibrionaceae* was higher in abundance in SW14 than FW14, especially in the posterior intestine (S4 Fig). The abundance of *Vibrionaceae* also increased from 0.83% in the posterior intestine of the FW14 condition to 22.10% in the posterior intestine of the SW14 condition (S4 Fig). There were also distinct patterns of intestinal zonation in the gut microbiome based on ANCOM level 5 for both the freshwater (S4 Fig) and seawater (S4 Fig) conditions. Particularly, in the SW condition, the abundance of *Vibrionaceae* was approximately twice as high in the posterior intestine (22.1%) than the anterior intestine (11.29%; S4 Fig).

**Fig 2:**
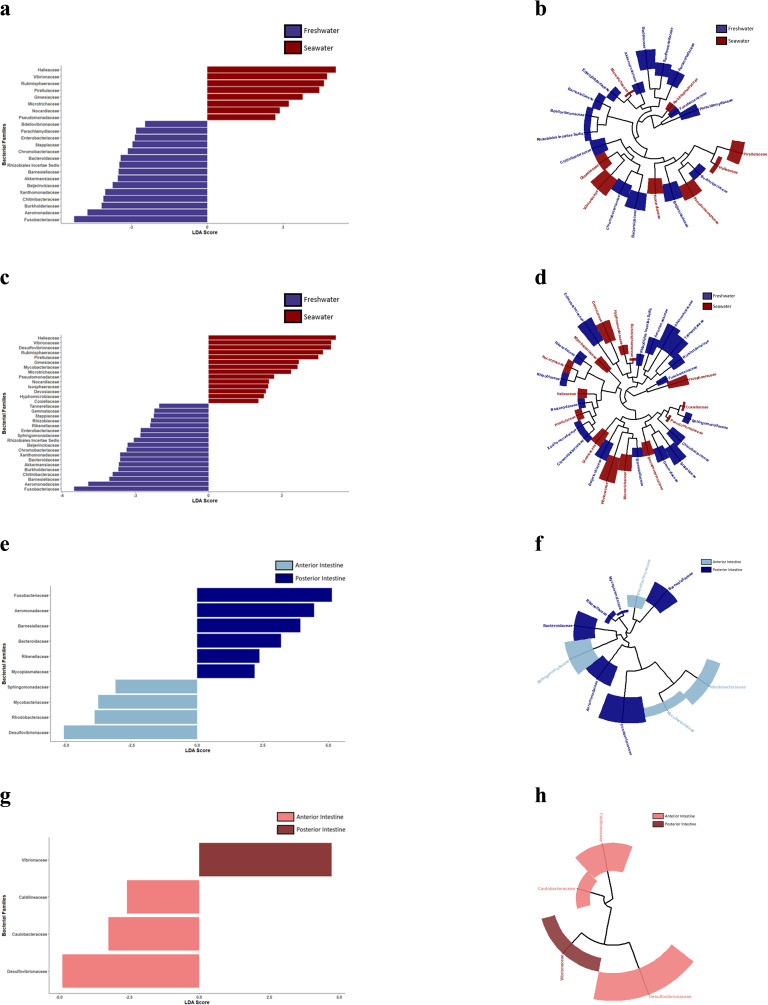
LDA of the gut microbiomes of freshwater and seawater sailfin mollies at the family level. The anterior (a) and posterior intestines (c) were compared across the freshwater and seawater environments and the respective cladograms (anterior intestines: b; posterior intestines: d) are also shown. Patterns of intestinal zonation in the gut microbiome of freshwater sailfin mollies (e) and seawater sailfin mollies (g) are plotted alongside the respective cladogram for each environment (freshwater: f; seawater: **h)**.

When the anterior intestines were directly compared between the two environments (freshwater and seawater) using LEfSE, *Bdellovibrionaceae, Parachlamydiaceae, Enterobacteriaceae, Stappiaceae, Chromobacteriaceae, Bacteroidaceae, Rhizobiales Incertae Sedis, Barnesiellaceae, Akkermansiaceae, Beijerinckiaceae, Xanthomonadaceae, Chitinibacteraceae, Burkholderiaceae, Aeromonadaceae,* and *Fusobacteriaceae* were more abundant in the freshwater condition while *Pseudomonadaceae, Nocardiaceae, Microtrichaceae, Gimesiaceae, Pirellulaceae, Rubinisphaeraceae, Vibrionaceae*, and *Halieaceae* were more abundant in the seawater condition (Fig 2a). The posterior intestines were directly compared between FW and SW environments using LEfSE and the following bacteria: *Tannerellaceae, Gemmataceae, Stappiaceae, Rhizobiaceae, Rikenellaceae, Enterobacteriaceae, Sphingomonadaceae, Rhizobiales Incertae Sedis, Beijerinckiaceae, Chromobacteriaceae, Xanthomonadaceae, Bacteroidaceae, Akkermansiaceae, Burkholderiaceae, Chitinibacteraceae, Barnesiellaceae, Aeromonadaceae,* and *Fusobacteriaceae* were more abundant in the freshwater condition while *Coxiellaceae, Hyphomicrobiaceae, Devosiaceae, Isosphaeraceae, Nocardiaceae, Pseudomonadaceae, Microtrichaceae, Mycobacteriaceae, Gimesiaceae, Pirellulaceae, Rubinisphaeraceae, Desulfovibrionaceae, Vibrionaceae,* and *Halieaceae* were more abundant in the seawater condition (Fig 2c). Analysis of the zonational patterns in the freshwater condition revealed that *Desulfovibrionaceae, Rhodobacteraceae, Mycobacteriaceae,* and *Sphingomonadaceae* were more abundant in anterior intestine while *Mycopiasmataceae, Rikenellaceae, Bacteroidaceae, Barnesiellaceae, Aeromonadaceae,* and *Fusobacteriaceae* were more abundant in the posterior intestine (Fig 2e). LEfSE also revealed zonational patterns in the seawater condition as *Desulfovibrionaceae, Caulobacteraceae,* and *Caldilineaceae* were more abundant in the anterior intestine while only *Vibrionaceae* was more abundant in the posterior intestine (Fig 2g). Phylogenetic cladograms were used to display the evolutionary relationships between the significant bacterial families identified through LEfSE between the freshwater and seawater conditions for the anterior intestines (Fig 2b) and posterior intestines (Fig 2d). Also, phylogenetic cladograms were used to display the evolutionary relationships between the significant bacterial families identified through LEfSE between the anterior and posterior intestines (patterns of zonation) for the freshwater (Fig 2f) and seawater (Fig 2h) conditions.

Several predicted KEGG Level 2 categories annotated under ‘Human Diseases’ were among the pathways identified as differentially abundant. These categories reflect KEGG database annotation groupings of microbial gene families and do not indicate disease processes in fish. Accordingly, these predictions are reported here to describe shifts in the inferred functional potential of the intestinal microbiome, rather than as evidence of pathological states.

**Fig 3:**
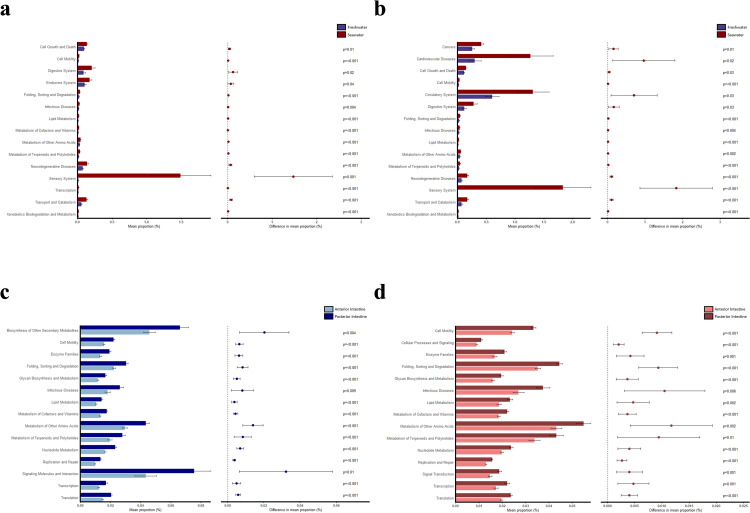
Predicted differential KEGG pathways across environmental salinities and intestinal sections. The top 15 (based on highest mean proportions) predicted differential KEGG pathways (Level 2 based on the full set of abundance data) between the freshwater and seawater environments for the anterior (a) and posterior (b) intestines; as well as intestinal zonation between the anterior and posterior intestines in freshwater (c) and seawater **(d)**. The bar plot on the left depicts each KEGG pathway’s mean proportion (%) and on the right, the difference in mean proportion (%) between the two groups, the 95% confidence interval, and p-value are shown.

In the anterior intestine, the top 15 (based on highest mean proportions) predicted differential KEGG pathways (Level 2 based on the full set of abundance data) were more abundant in seawater than freshwater: cell growth and death; cell motility; digestive system; endocrine system; folding, sorting, and degradation; infectious diseases; lipid metabolism; metabolism of cofactors and vitamins; metabolism of other amino acids; metabolism of terpenoids and polyketides; neurodegenerative diseases; sensory system; transcription; transport and catabolism; and xenobiotics biodegradation and metabolism (Fig 3a). In the posterior intestine, the top 15 (based on highest mean proportions) predicted differential KEGG pathways (Level 2 based on the full set of abundance data) were more abundant in seawater than freshwater: cancers; cardiovascular diseases; cell growth and death; cell motility; circulatory system; digestive system; folding, sorting, and degradation; infectious diseases; lipid metabolism; metabolism of other amino acids; metabolism of terpenoids and polyketides; neurodegenerative diseases; sensory system; transport and catabolism; and xenobiotics biodegradation and metabolism (Fig 3b).

In the freshwater condition, the top 15 (based on highest mean proportions) predicted differential KEGG pathways (Level 2 based on the full set of abundance data) were more abundant in posterior intestine than anterior intestine: biosynthesis of other secondary metabolites; cell motility; enzyme families; folding, sorting, and degradation; glycan biosynthesis and metabolism; infectious diseases; lipid metabolism; metabolism of cofactors and vitamins; metabolism of other amino acids; metabolism of terpenoids and polyketides; nucleotide metabolism; replication and repair; signaling molecules and interaction; transcription; and translation (Fig 3c). In the seawater condition, the top 15 (based on highest mean proportions) predicted differential KEGG pathways (Level 2 based on the full set of abundance data) were more abundant in posterior intestine than anterior intestine: cell motility; cellular processes and signaling; enzyme families; folding, sorting, and degradation; glycan biosynthesis and metabolism; infectious diseases; lipid metabolism; metabolism of cofactors and vitamins; metabolism of other amino acids; metabolism of terpenoids and polyketides; nucleotide metabolism; replication and repair; signal transduction; transcription; and translation (Fig 3d).

In the anterior intestine, the following predicted differential KEGG pathways (Level 2) under the metabolism category were higher in seawater than freshwater: amino acid metabolism; carbohydrate metabolism; energy metabolism; enzyme families; glycan biosynthesis and metabolism; lipid metabolism; metabolism of cofactors and vitamins; metabolism of other amino acids; metabolism of terpenoids and polyketides; nucleotide metabolism; and xenobiotics biodegradation and metabolism (S5 Fig). In the posterior intestine, the following predicted differential KEGG pathways (Level 2) under the metabolism category were higher in seawater than freshwater: amino acid metabolism; carbohydrate metabolism; energy metabolism; glycan biosynthesis and metabolism; lipid metabolism; metabolism of cofactors and vitamins; metabolism of other amino acids; metabolism of terpenoids and polyketides; and xenobiotics biodegradation and metabolism (S5 Fig). In freshwater, the following predicted differential KEGG pathways (Level 2) under the metabolism category were higher in the posterior intestine than the anterior intestine: amino acid metabolism; biosynthesis of other secondary metabolites; carbohydrate metabolism; energy metabolism; enzyme families; glycan biosynthesis and metabolism; lipid metabolism; metabolism of cofactors and vitamins; metabolism of other amino acids; metabolism of terpenoids and polyketides; nucleotide metabolism; and xenobiotics biodegradation and metabolism (S5 Fig). In seawater, the following predicted differential KEGG pathways (Level 2) under the metabolism category were higher in the posterior intestine than the anterior intestine: amino acid metabolism; carbohydrate metabolism; energy metabolism; enzyme families; glycan biosynthesis and metabolism; lipid metabolism; metabolism of cofactors and vitamins; metabolism of other amino acids; metabolism of terpenoids and polyketides; and nucleotide metabolism (S5 Fig).

In the anterior intestines and posterior intestines, the top 15 (based on highest mean proportions) predicted differential KEGG pathways (Level 3) under the metabolism category were compared across the freshwater and seawater conditions (S5 Fig). In the anterior intestine, the following predicted KEGG pathways (Level 3 of Metabolism Category) were more abundant in seawater than freshwater: betalain biosynthesis; biosynthesis of type II polyketide products; caprolactam degradation; d-arginine and d-ornithine metabolism; glycosaminoglycan degradation; glycosphingolipid biosynthesis – ganglio series; indole alkaloid biosynthesis; limonene and pinene degradation; lipoic acid metabolism; other types of o-glycan biosynthesis; primary bile acid biosynthesis; steroid biosynthesis; steroid hormone biosynthesis; and synthesis and degradation of ketone bodies while isoflavonoid biosynthesis was more abundant in freshwater than seawater (S5 Fig). In the posterior intestine, the following predicted KEGG pathways (Level 3 of Metabolism Category) were more abundant in seawater than freshwater: betalain biosynthesis; biosynthesis of type II polyketide products; caffeine metabolism; cytochrome P450; d-arginine and d-ornithine metabolism; glycosphingolipid biosynthesis – ganglio series; glycosylphosphatidylinositol (GPI)-anchor biosynthesis; indole alkaloid biosynthesis; and other types of O-glycan biosynthesis while biosynthesis of vancomycin group antibiotics; flavone and flavonol biosynthesis; glycosaminoglycan biosynthesis – chondroitin sulfate; isoflavonoid biosynthesis; secondary bile acid biosynthesis; and stilbenoid, diarylheptanoid, and gingerol biosynthesis (S5 Fig).

The top 15 (based on highest mean proportions) predicted differential KEGG pathways (Level 3) under the metabolism category were compared between the anterior and posterior intestines to elucidate patterns of intestinal zonation in the freshwater and seawater conditions (S5 Fig). In the freshwater condition, the following predicted KEGG pathways (Level 3 of Metabolism Category) were more abundant in the posterior intestine than the anterior intestine: biosynthesis of vancomycin group antibiotics; d-glutamine and d-glutamate metabolism; drug metabolism – other enzymes; galactose metabolism; glutathione metabolism; glycerolipid metabolism; glycosphingolipid biosynthesis – ganglio series; isoflavonoid biosynthesis; other glycan degradation; secondary bile acid biosynthesis; selenocompound metabolism; streptomycin biosynthesis; and tetracycline biosynthesis while betalain biosynthesis and polycyclic aromatic hydrocarbon degradation was more abundant in the anterior intestine than posterior intestine (S5 Fig). In the seawater condition, the following predicted KEGG pathways (Level 3 of Metabolism Category) were more abundant in the posterior intestine than the anterior intestine: amino sugar and nucleotide sugar metabolism; biosynthesis of vancomycin group antibiotics; citrate cycle (TCA cycle); folate biosynthesis; glutathione metabolism; glycine, serine, and threonine metabolism; glycosphingolipid biosynthesis – ganglio series; lipopolysaccharide biosynthesis proteins, nicotinate and nicotinamide metabolism; pentose phosphate pathway; protein kinases; pyruvate metabolism; secondary bile acid biosynthesis; ubiquinone and other terpenoid-quinone biosynthesis; and valine, leucine, and isoleucine biosynthesis (S5 Fig).

**Fig 4:**
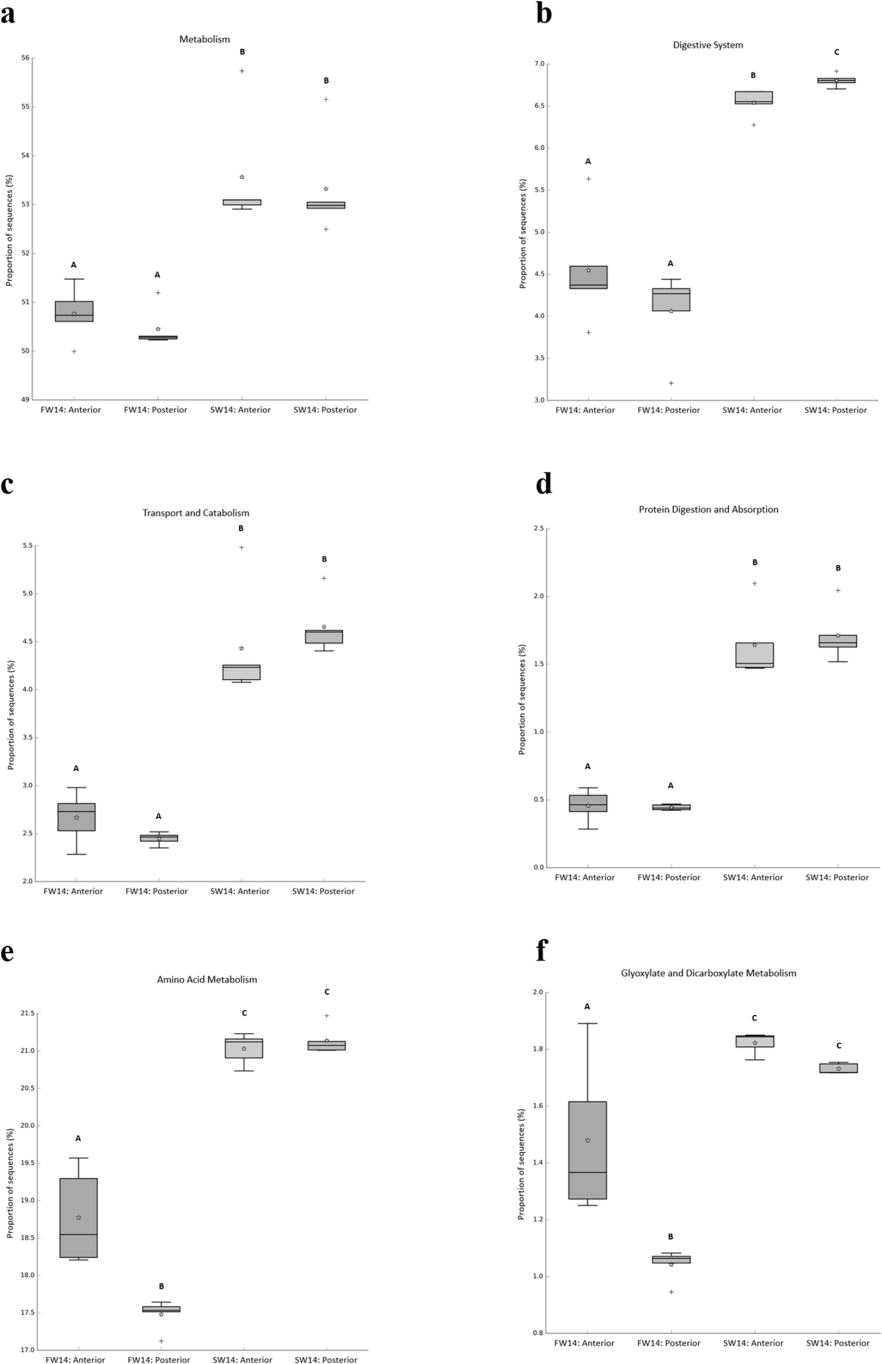
Predicted functional differences in the gut microbiome of freshwater and seawater sailfin mollies are shown, with significant variations observed in the proportion of sequences (%) for several key metabolic functions. Significant differences were observed in the proportion of sequences (%) for the following functions: metabolism (a); digestive system (b); transport and catabolism (c); protein digestion and absorption (d); amino acid metabolism (e); and glyoxylate and dicarboxylate metabolism (f). The star symbols (∂) represent the mean of each group and the addition symbols (+) represent outliers. Each of the four groups (FW14-Anterior, FW14-Posterior, SW14-Anterior, SW-14-Posterior) have an n = 5. A two-way ANOVA with Tukey post-hoc test was used for each panel. Within each panel, groups that share letters are not significantly different.

Based on PICRUSt2 predictive functional profiling, there were significant differences in the predictive functions of the gut microbiome in sailfin mollies acclimated to either freshwater or seawater (Fig 4). Particularly, the following predictive functions of the gut microbiome were increased in seawater sailfin mollies than their counterparts in freshwater: overall metabolism (Fig 4a), digestive system (Fig 4b), transport and catabolism (Fig 4c), protein digestion and absorption (Fig 4d), amino acid metabolism (Fig 4e), as well as glyoxylate and dicarboxylate metabolism (Fig 4f). Overall, the predictive microbial functions of metabolism (Fig 4a), transport and catabolism (Fig 4c) and protein digestion and absorption (Fig 4d) were more abundant in the seawater conditions but did not show any patterns of intestinal zonation. The following predictive functions: digestive system (Fig 4b), amino acid metabolism (Fig 4e), and glyoxylate and dicarboxylate metabolism (Fig 4f) of the gut microbiome were higher in the seawater than freshwater conditions and also showed patterns of intestinal zonation in either freshwater, seawater, or both environments.

### Salinity exposure: Total, free, and precipitated oxalate concentrations

**Fig 5:**
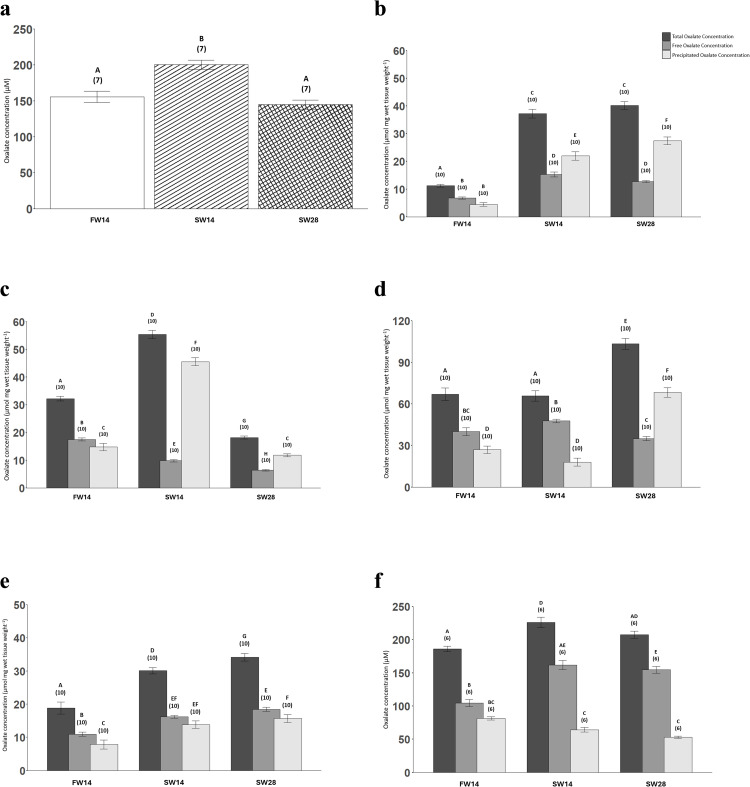
Oxalate concentrations in the plasma (µM;a), and in the following tissues (µmol mg wet tissue weight^-1^): anterior intestines (b), posterior intestines (c), and kidneys (d), liver (e), and urine (f) across the FW14, SW14, and SW28 conditions. The total, free, and precipitated oxalate concentrations are represented by black, dark grey, and light grey bars respectively. The bars represent column means ± SEM (n-values). Within each panel, bars that share letters are not statistically different. For panels a, d, e, and f, mixed-effects linear model regressions were employed. For panels b and c, the datasets did not pass normality and generalized additive models were used. In all analyses, individual fish were treated as the unit of observation, and tank identity and sex were included in the models to account for potential clustering; no significant tank or sex effects were detected, so fish were treated as independent observations for inference.

When acclimated to freshwater, the plasma oxalate concentration was 155.56 ± 7.90 µM (Fig 5a). After acclimating to seawater for 14 days, plasma oxalate concentrations showed a transient increase of nearly 50% before decreasing to values similar to freshwater when seawater acclimation was extended to 28 days (Fig 5a).

For the FW14 condition, the total oxalate concentration of the anterior intestine was 11.25 ± 0.52 µmol mg wet tissue weight^-1^ (Fig 5b). When acclimated to seawater for 14 and 28 days, the total oxalate concentrations of the anterior intestine were 3.7-fold and 4-fold higher respectively than in freshwater (Fig 5b). Similar to the anterior intestine, the total oxalate concentration of the posterior intestine increased in response to short-term salinity from 32.26 ± 0.83 µmol mg wet tissue weight^-1^ in freshwater to 55.44 ± 1.47 µmol mg wet tissue weight^-1^ (Fig 5c). However, after long-term seawater acclimation, the total oxalate concentration of the posterior intestine decreased to approximately half of what was observed in freshwater (Fig 5c).

Acclimation to seawater for 14 days more than doubled the free oxalate concentration of the anterior intestine when compared to freshwater acclimation (15.29 ± 0.91 vs. 6.81 ± 0.41 µmol mg wet tissue weight^-1^; Fig 5b). This rise in concentration was reduced after 28 days in seawater though, with free oxalate concentrations falling to an intermediate level between both freshwater and 14-day seawater acclimated concentrations (Fig 5b). In contrast, the free oxalate concentrations in the posterior intestine decreased with seawater exposure (Fig 5c). Indeed, acclimating to seawater for 14 days nearly halved the free oxalate concentration compared to freshwater (decreasing from 17.50 ± 0.54 to 9.84 ± 0.46 µmol mg wet tissue weight^-1^) before it was reduced another ~30% following 28 days in seawater (6.42 ± 0.27 µmol mg wet tissue weight^-1^; Fig 5c).

The precipitated oxalate concentration in the anterior intestine increased in response to salinity from 4.44 ± 0.63 µmol mg wet tissue weight^-1^ in freshwater to 5-fold higher after seawater acclimation for 14 days and remained elevated when seawater acclimation was extended to 28 days (Fig 5b). Similar to the anterior intestine, the precipitated oxalate concentration in the posterior intestine initially increased in response to salinity from 14.76 ± 1.3 µmol mg wet tissue weight^-1^ in freshwater to triple the concentration after seawater acclimation for 14 days but decreased to levels akin to freshwater after long-term salinity exposure (Fig 5c).

In freshwater, there was no statistical difference between the proportions of free (60.5%) and precipitated (39.5%) oxalate but in the short-term salinity acclimation the precipitated oxalate (59.0%) was significantly higher than the free oxalate (41.0%) in the anterior intestine (Fig 5b). The trend was also apparent in the anterior intestine after the long-term salinity acclimation where the precipitated oxalate (68.3%) was still greater than the free oxalate (31.7%; Fig 5b). In freshwater, the free oxalate (54.3%) was greater than the precipitated oxalate (45.7%) in the posterior intestine (Fig 5c). However, the free oxalate (17.7%) was much lower than the precipitated oxalate (82.3%) in the posterior intestine of the SW14 condition (Fig 5c). This trend in the posterior intestine remained in the SW28 condition with the free oxalate (35.3%) being much lower than the precipitated oxalate (64.8%; Fig 5c).

Short-term salinity acclimation did not alter the total oxalate of the kidney as the total oxalate concentration of the kidney was 67.03 ± 4.49 µmol mg wet tissue weight^-1^ in freshwater and remained unchanged after both short-term and long-term salinity exposure (Fig 5d). However, long-term salinity acclimation did result in an increase of the total oxalate concentration in the kidney to 103.37 ± 4.01 µmol mg wet tissue weight^-1^ (Fig 5d). The free oxalate concentration in the kidney initially increased due to short-term salinity acclimation (from 40.02 ± 2.93 to 47.72 ± 1.28 µmol mg wet tissue weight^-1^) but long-term salinity exposure (35.03 ± 1.47 µmol mg wet tissue weight^-1^) lowered the free oxalate concentration back down to levels observed in freshwater (Fig 5d). Similar to the total oxalate concentration in the kidney, the precipitated oxalate concentrations were not altered due to short-term salinity acclimation with the precipitated oxalate concentration being 27.01 ± 2.65 µmol mg wet tissue weight^-1^ in freshwater and 18.04 ± 2.83 µmol mg wet tissue weight^-1^ in the SW14 conditions (Fig 5d). However, long-term salinity exposure resulted in a 2.5-fold increase in the precipitated oxalate concentration in the kidney to 68.34 ± 3.47 µmol mg wet tissue weight^-1^ (Fig 5d).

In FW14, the free oxalate of the kidney (59.7%) was significantly higher than the precipitated oxalate (40.3%; Fig 5d). This trend remained for the kidney in SW14 with the free oxalate (72.6%) higher than the precipitated oxalate (27.4%; Fig 5d). However, after long-term salinity acclimation, the precipitated oxalate (66.1%) in the kidney was higher than the free oxalate (33.9%; Fig 5d).

The total oxalate concentration of the liver in freshwater was 18.85 ± 1.84 µmol mg wet tissue weight^-1^ (Fig 5e) and nearly doubled due to short-term salinity and remained elevated when seawater acclimation was extended to 28 days (Fig 5e). The free oxalate concentration in the liver was 10.98 ± 0.64 µmol mg wet tissue weight^-1^ in freshwater and increased 1.5-fold after seawater acclimation for 14 days (Fig 5e). The free oxalate concentration further increased in the long-term salinity acclimation of 28 days and was nearly double the level of the freshwater condition (Fig 5e). The precipitated oxalate concentrations in the liver of both the short-term and long-term seawater conditions were approximately double the levels observed in the freshwater condition (7.87 ± 1.36 µmol mg wet tissue weight^-1^; Fig 5e).

In freshwater, the free oxalate (58.2%) in the liver was higher than the precipitated oxalate (41.8%; Fig 5e). However, after short-term salinity acclimation there was no statistically significant difference between the free oxalate (54.0%) and the precipitated oxalate (46.0%) in the liver (Fig 5e). Long-term salinity acclimation resulted in the free oxalate (54.1%) of the liver being higher than the precipitated oxalate (45.9%; Fig 5e).

In freshwater, the total oxalate concentration in the urine was 186.0 ± 4.02 µM, with free and precipitated oxalate concentrations of 105.0 ± 5.39 µM and 81.5 ± 2.66 µM, respectively (Fig 5f). After 14 days of seawater acclimation, total urinary oxalate increased to 226.0 ± 7.69 µM, driven primarily by a rise in free oxalate to 162.0 ± 6.92 µM, while precipitated oxalate decreased to 64.3 ± 3.64 µM (Fig 5f). After 28 days in seawater, total urinary oxalate decreased slightly to 208.0 ± 5.34 µM, with free oxalate remaining elevated at 155.0 ± 5.27 µM and precipitated oxalate further decreasing to 53.1 ± 1.77 µM (Fig 5f). In freshwater, the proportion of free oxalate (56.5%) was greater than precipitated oxalate (43.5%; Fig 5f). Short-term seawater acclimation shifted this balance, with free oxalate (71.7%) predominating over precipitated oxalate (28.3%), and the trend persisted during long-term seawater exposure (free: 74.5%, precipitated: 25.5%; Fig 5f). These changes in the urine were consistent, where total and free oxalate in SW14 and SW28 were significantly higher than FW14, while precipitated oxalate decreased significantly with salinity.

### Salinity exposure: SLC26A3 and SLC26A6 gene expression

**Fig 6:**
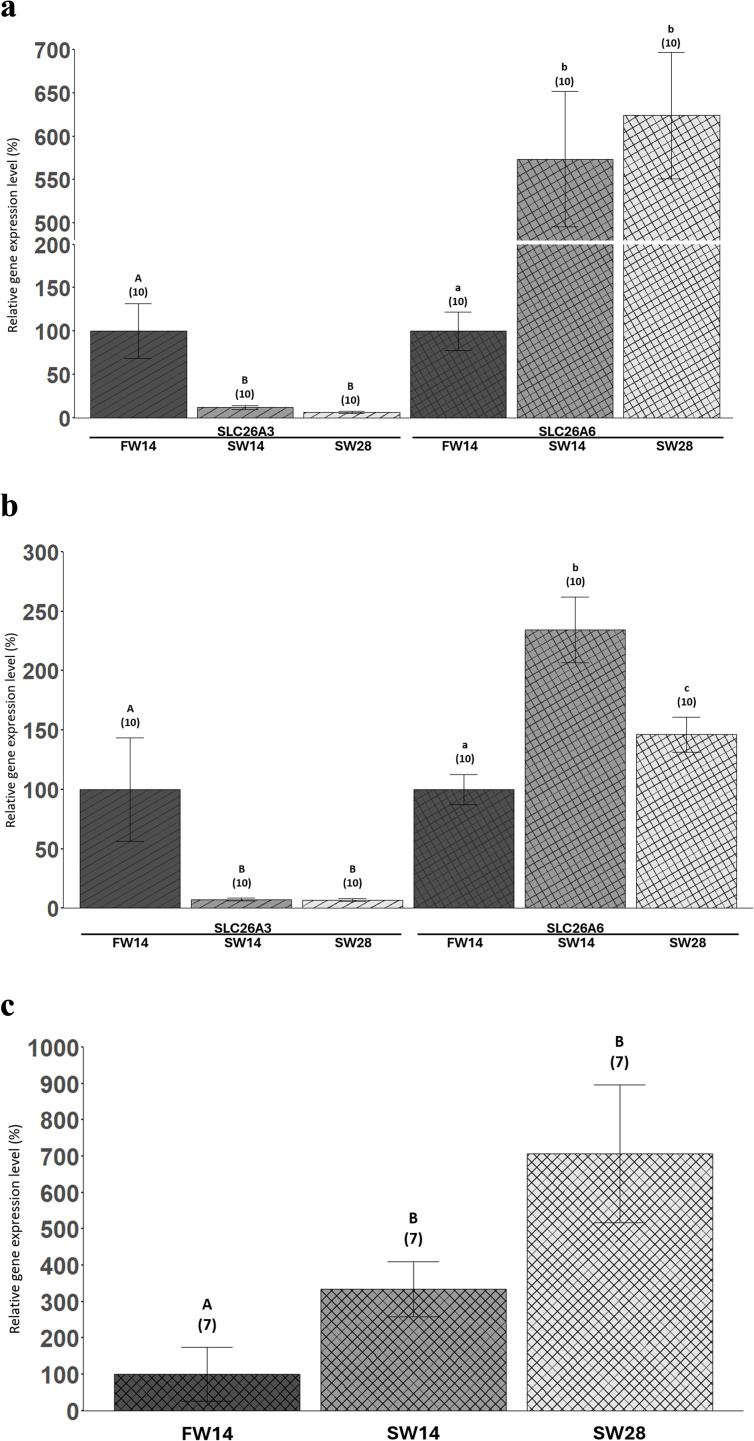
SLC26A3 (striped bars) and SLC26A6 (cross-hatched bars) gene expression levels (%) in the anterior intestines (a), posterior intestines (b), and kidneys (c) across the FW14, SW14, and SW28 conditions. The relative gene expression levels are expressed as percentages with the control (FW14) condition set to 100%. The bars represent column means ± SEM (n-values). Within each panel, bars that share symbols are not statistically different. For SLC26A3, datasets in both the anterior and posterior intestines (a & b) did not pass normality and generalized additive models were used. For SLC26A6 in the anterior intestine **(a)**, all model assumptions were met, and a mixed-effects linear model regression was used. For SLC26A6, datasets in the posterior intestine (b) and kidney (c) were log(10)-transformed to meet normality and then mixed-effects linear model regressions were employed. In all analyses, individual fish were treated as the unit of observation, and tank identity and sex were included in the models to account for potential clustering; no significant tank or sex effects were detected, so fish were treated as independent observations for inference.

SLC26A3 expression was undetectable in the kidneys. SLC26A3 expression in the anterior intestine for the short-term and long-term seawater acclimation were 88% and 94% lower than the freshwater condition respectively (Fig 6a). Further, SLC26A3 expression in the posterior intestine for the short-term and long-term seawater acclimation were both ~93% lower than freshwater (Fig 6b). There was no difference in SLC26A3 gene expression between the short-term and long-term seawater conditions in both the anterior and posterior intestines (Figs 6a & 6b).

SLC26A6 expression in the anterior intestine increased by 5.7-fold and 6.2-fold in the short-term and long-term seawater acclimation respectively when compared to the anterior intestine of the freshwater condition (Fig 6a). Akin to SLC26A3 expression, when SLC26A6 expression in the anterior intestine of both the short-term and long-term seawater conditions were compared, there was no difference (Fig 6a). SLC26A6 expression in the posterior intestine was 2.3-fold higher in the short-term salinity acclimation than the freshwater condition (Fig 6b). SLC26A6 expression in the posterior intestine of the long-term salinity acclimation was 1.5-fold higher than the freshwater condition but lower than the short-term salinity exposure (Fig 6b). After short-term seawater acclimation, renal SLC26A6 expression increased 3.3-fold from the freshwater condition and remained elevated when seawater acclimation was extended to 28 days (Fig 6c).

### Antibiotic exposure: Total, free, and precipitated oxalate concentrations

**Fig 7:**
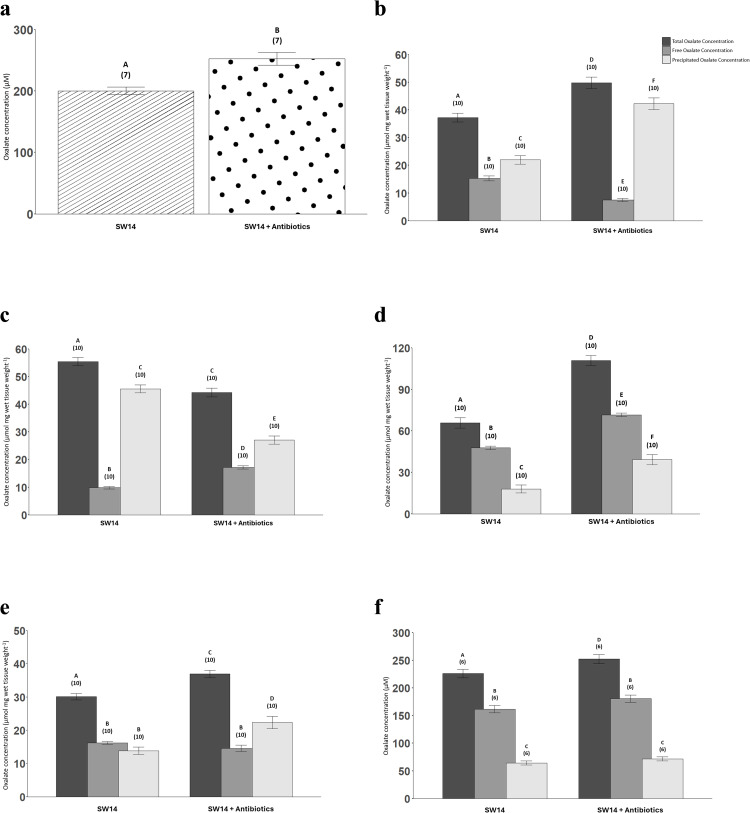
Oxalate concentrations in the plasma (µM; a), and in the following tissues (µmol mg wet tissue weight^-1^): anterior intestines (b), posterior intestines (c), and kidneys (d), liver (e), and urine (f) in both the control and antibiotic treatment conditions. Both the control and antibiotic exposures were run for 14 days in seawater. The total, free, and precipitated oxalate concentrations are represented by black, dark grey, and light grey bars respectively. The bars represent column means ± SEM (n-values). Within each panel, bars that share letters are not statistically different. For panels a, d, e, and f, mixed-effects linear model regressions were utilized. For panels b and c, the datasets did not pass normality and thus, generalized additive models were used. In all analyses, individual fish were treated as the unit of observation, and tank identity and sex were included in the models to account for potential clustering; no significant tank or sex effects were detected, so fish were treated as independent observations for inference.

The plasma oxalate concentration was 200.37 ± 6.23 µM after seawater acclimation for 14 days and increased ~25% due to antibiotic exposure (Fig 7a).

The total oxalate concentration in the anterior intestine was 37.28 ± 1.58 µmol mg wet tissue weight^-1^ in the SW14 condition and increased ~40% in the SW14 + Antibiotics condition (Fig 7b). Contrastingly, the total oxalate concentration in the posterior intestine was 55.44 ± 1.47 µmol mg wet tissue weight^-1^ in the SW14 condition and decreased ~20% due to broad-range antibiotic treatment (Fig 7c).

The free oxalate concentration in the anterior intestine was 15.29 ± 0.91 µmol mg wet tissue weight^-1^ in the SW14 condition and halved when treated with broad-range antibiotics for 14 days (Fig 7b). In contrast, the free oxalate concentration of the posterior intestine was 9.84 ± 0.46 µmol mg wet tissue weight^-1^ in the seawater acclimation for 14 days and nearly doubled when treated with broad-range antibiotics for two weeks (Fig 7c).

The precipitated oxalate concentrations in the anterior and posterior intestines exposed to antibiotics follow a similar pattern to that of the total oxalate concentrations, with the precipitated oxalate concentration increasing due to antibiotic treatment in the anterior intestine but decreasing in the posterior intestine. In the anterior intestine, the precipitated oxalate was 21.98 ± 1.52 µmol mg wet tissue weight^-1^ in SW14 and doubled when treated with broad-spectrum antibiotics (Fig 7b). Further, in the posterior intestine, the precipitated oxalate concentration was 45.60 ± 1.44 µmol mg wet tissue weight^-1^ in SW14 and decreased by ~40% when treated with broad-range antibiotics for 14 days (Fig 7c).

As expected, antibiotic exposure increased the oxalate concentrations in the kidney. For the SW14 condition, the total oxalate concentration of the kidney was 65.76 ± 3.80 mg wet tissue^-1^ and the levels increased by ~70% when fish were treated with antibiotics (Fig 7d). The free oxalate concentration was 47.72 ± 1.28 µmol mg wet tissue weight^-1^ in the kidney of sailfin mollies acclimated to seawater for 14 days and renal concentrations increased by ~50% when these fish were treated with broad-range antibiotics for two weeks (Fig 7d). Also, the precipitated oxalate concentration was 18.04 ± 2.83 µmol mg wet tissue weight^-1^ in the SW14 condition and increased ~120% in the broad-range antibiotic exposure (Fig 7d).

Antibiotic exposure resulted in an increase in the total oxalate concentration of the liver from 30.11 ± 0.95 mg wet tissue^-1^ in the SW14 condition to 36.94 ± 1.14 mg wet tissue^-1^ in the SW14 + Antibiotics condition (Fig 7e). Further, the free oxalate concentrations in the liver remained unchanged after antibiotic exposure (Fig 7e). However, the precipitated oxalate concentration in the liver increased ~60% due to antibiotics as the precipitated oxalate concentrations were 13.87 ± 1.12 µmol mg wet tissue weight^-1^ and 22.36 ± 1.88 µmol mg wet tissue weight^-1^ in the SW14 and SW14 + Antibiotics conditions respectively (Fig 7e).

In the anterior intestine of the SW + Antibiotics condition, the free oxalate (15.0%) was much lower than the precipitated oxalate (85.0%; Fig 7b). A similar pattern was observed in the posterior intestine after antibiotic treatment as the free oxalate (38.9%) was lower than the precipitated oxalate (61.1%; Fig 7c). However, the kidney showed a different trend as after antibiotic treatment the free oxalate (64.6%) was greater than the precipitated oxalate (35.4%; Fig 7d). The liver showed an opposite pattern to the kidney, as the free oxalate (39.5%) was lower than the precipitated oxalate (60.5%; Fig 7e).

### Antibiotic exposure: SLC26A3 and SLC26A6 gene expression

**Fig 8:**
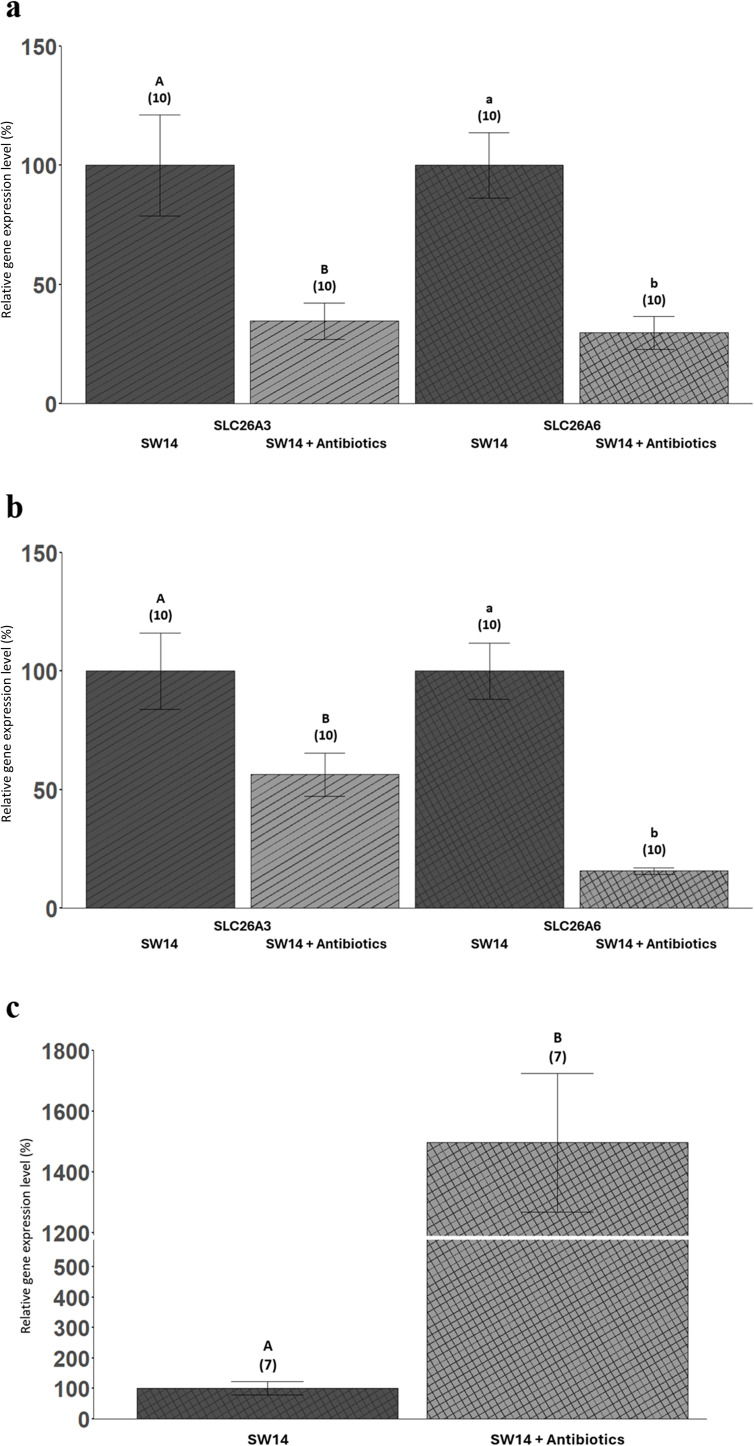
SLC26A3 (striped bars) and SLC26A6 (cross-hatched bars) gene expression levels (%) in the anterior intestines(a), posterior intestines (b), and kidneys (c) in both the control and antibiotic treatment. Both the control and antibiotic exposures were run for 14 days in seawater. The relative gene expression levels are expressed as percentages with the control (SW14) condition set to 100%. The bars are means ± SEM (n-values). Within each panel, bars that share symbols are not statistically different. For SLC26A3, the dataset for the anterior intestine (a) was log(10)-transformed to meet normality and subsequently a mixed-effects linear model regression was used. For the posterior intestine **(b)**, the SLC26A3 dataset did not require any transformation prior to utilizing a mixed-effects linear model regression. For the anterior intestine **(a)**, the SLC26A6 dataset did not require any transformation prior to using a mixed-effects linear model regression. For SLC26A6, the datasets for the posterior intestine (b) and kidney (c) were log(10)-transformed to meet normality and mixed-effects linear model regressions were used. In all analyses, individual fish were treated as the unit of observation, and tank identity and sex were included in the models to account for potential clustering; no significant tank or sex effects were detected, so fish were treated as independent observations for inference.

When SLC26A3 expression was directly compared in the anterior intestines of the SW14 condition and SW14 + Antibiotics condition, a 65% decrease in SLC26A3 gene expression was observed due to antibiotic treatment (Fig 8a). Also, when SLC26A3 expression was directly compared in the posterior intestines of the SW14 and SW14 + Antibiotics conditions, SLC26A3 was found to be 44% lower in the broad-range antibiotic treatment condition (Fig 8b).

Unexpectedly, the effects of the broad-range antibiotics on SLC26A6 expression were similar to the impacts observed for SLC26A3. Particularly, SLC26A6 expression in the anterior intestine of the SW14 + Antibiotics condition was 70% lower than the SW14 condition (Fig 8a). Likewise, SLC26A6 expression in the posterior intestines was 84% lower in the SW14 + Antibiotics condition than the SW14 condition (Fig 8b). The gene expression level of SLC26A6 was nearly 15-fold higher in the kidneys of seawater sailfin mollies treated with broad-range antibiotics for two weeks than their counterparts in the SW14 condition (Fig 8c).

In the SW14 + Antibiotics condition, total urinary oxalate was ~ 25 µM higher in the SW14 + Antibiotics condition (252.0 ± 8.12 µM), compared to 226.0 ± 7.69 µM in SW14 without antibiotics (Fig 7f). The free oxalate concentration in the urine was 162.0 ± 6.92 µM and 181.0 ± 6.58 µM in the SW14 and SW14 + Antibiotics respectively (Fig 7f). The precipitated oxalate concentration in the urine was 71.8 ± 3.83 µM in the SW14 + Antibiotics condition and 64.3 ± 3.64 µM in SW14 (Fig 7f). However, none of these differences for the free and precipitated oxalate concentrations were statistically significant (Fig 7f). The proportions of free and precipitated oxalate in the urine for the SW14 + Antibiotics condition mirrored those in SW14, with free oxalate predominating (~72%) over precipitated oxalate (~28%; Fig 7f).

### Intestinal zonation for salinity and antibiotics exposure

Surprisingly, there were clear patterns of zonation of oxalate concentrations along the intestine in the FW14, SW14, and SW28 conditions. The total oxalate concentration was higher in the posterior intestine than the anterior intestine for the FW14 (S2 Fig) and SW14 (S2 Fig) conditions, but the opposite was seen for the SW28 (S2 Fig) condition. The free oxalate concentration was higher in the anterior intestine than the posterior intestine for the seawater conditions (SW14 and SW28; S2 Fig) but the opposite was observed for the FW14 condition (S2 Fig). The precipitated oxalate concentration was higher in the posterior intestine than the anterior intestine for the FW14 and SW14 conditions, but the opposite pattern of zonation was observed in the SW28 condition (S2 Fig).

Furthermore, there were clear patterns of zonation of oxalate concentrations along the intestine for the SW14 + Antibiotics condition. Both the total and precipitated oxalate concentrations were higher in the anterior intestine than the posterior intestine (S2 Fig). However, the opposite was observed for the free oxalate concentration in the seawater sailfin mollies that were treated with the broad-spectrum antibiotics as the free oxalate concentration was higher in the posterior intestine than the anterior intestine (S2 Fig).

Zonation of SLC26A3 gene expression along the intestine was analogous regardless of treatment (FW14, SW14, SW28, SW14 + Antibiotics). Particularly, SLC26A3 expression was reduced in the posterior intestines of the FW14 condition (S3 Fig) indicating a decrease in expression along the intestine. SLC26A3 gene expression similarly decreased along the intestine when fish were acclimated to both short-term (SW14; S3 Fig) and long-term (SW28; S3 Fig) salinity. Antibiotic exposure did not alter the pattern of zonation, as gene expression of SLC26A3 decreased nearly 75% along the intestine (S3 Fig), similar to the other conditions.

Unlike the intestinal zonation of SLC26A3, when SLC26A6 expression was directly compared between the anterior and posterior sections of the intestine within the FW14 condition, there was no statistically significant difference in expression levels (S3 Fig). However, SLC26A6 zonation along the intestine of the SW14, SW28, and SW14 + Antibiotics conditions indicated a similar decreasing pattern along the intestine that was observed with SLC26A3 (S3 Fig).

## Discussion

Our findings indicate that environmental salinity influences the microbiome composition with the intestine as well as oxalate metabolism in *P. latipinna*, affecting oxalate production in tissues, its transport via renal and intestinal routes, and the possible role of the gut microbiome in homeostatic regulation.

The abundance of *Vibrionaceae* was elevated (Fig 2a–2c) in both the anterior (FW: 0.38% vs. SW: 11.29%; S4 Fig) and posterior intestine (FW: 0.83% vs SW: 22.1%; S4 Fig) while *Desulfovibrionaceae* was more abundant in the posterior intestine only (Fig 2c). *Vibrio* species are typically fast-growing, facultative anaerobes that thrive in high-salt, high-nutrient conditions [79,80,81], reviewed by [82] and are associated with marine environments [83,84,85], reviewed by [86]. While oxalate metabolism has not been extensively studied in this group, evidence suggests that some members of *Vibrionaceae* may have oxalate-degrading capabilities [87]. Thus, their presence could potentially influence oxalate handling, either directly through putative degradation or indirectly by altering microbial community structure.

As with *Vibrionaceae*, species from *Desulfovibrionaceae* are also able to degrade oxalate [88,89], highlighting a potential role for oxalate degradation in the posterior intestine specifically.

In conjunction, predictive functions of the gut microbiome were elevated in SW compared to FW (overall metabolism, digestive system, transport and catabolism, protein digestion and absorption, amino acid metabolism, as well as glyoxylate and dicarboxylate metabolism; Fig 4). It is important to note that PICRUSt2 provides predictions of functional potential based on 16S rRNA gene profiles and reference genomes, and does not measure gene expression or metabolic activity directly. Furthermore, KEGG pathway categories labeled under ‘Human Diseases’ represent database annotation groupings of microbial gene families rather than biologically meaningful disease processes in this system. Therefore, these predictions are interpreted here only as indicators of shifts in underlying functional and metabolic potential of the gut microbiome, with emphasis placed on metabolism- and transport-related pathways.

When examined together, the increased abundance of *Vibrionaceae* (anterior and posterior intestines: Figs 2a & 2c) and *Desulfovibrionaceae* (posterior intestine only: Fig 2c), along with the upregulation of glyoxylate and dicarboxylate metabolism in the gut microbiome (Fig 4f), are consistent with a potential role for microbial activity in intestinal oxalate processing. Given that intestinal oxalate measurements represent a composite of tissue, luminal contents, and precipitated forms, it remains unclear which compartment is most affected. Nonetheless, these findings are consistent with a microbial contribution to oxalate handling in a marine environment where urinary excretion is limited. However, these functional inferences are based on PICRUSt2 predictions from 16S rRNA gene data and therefore reflect putative metabolic potential rather than direct measurements of microbial activity or oxalate degradation. Furthermore, bacteria from the gut microbiome of these fish cannot be cultured or isolated, precluding direct experimental validation of their functional properties, including metabolic and oxalate-degrading capabilities.

Our findings underscore that the plasticity and “zonation” of oxalate-degrading bacteria are important factors in teleost metabolic physiology beyond nutrition. Under stable conditions, the posterior microbiota of *P. latipinna* appeared to be associated with low distal oxalate levels, consistent with efficient oxalate removal — FW control fish, for instance, showed only trace amounts of oxalate reaching the rectum, despite measurable production in tissues and presumably dietary intake (S2 Fig). In SW fish, despite a higher oxalate load entering the gut, which may result from increased secretion via transporters (S3 Fig), the posterior microbiome composition shifted in a manner consistent with increased oxalate handling, as evidenced by an initial moderate rise in oxalate concentrations before a reduction to below freshwater levels (Fig 5c; S2 Fig).

This speaks to a degree of functional plasticity of oxalate-degrading bacteria: the posterior microbiome can respond to greater oxalate availability (or other luminal changes) through shifts consistent with increased predicted activity (Fig 4f) or abundance of putative oxalate-degrading populations (specific species from bacterial families (*Vibrionaceae* and *Desulfovibrionaceae*) have oxalate-degrading properties: Figs 2c, 4f; S4 Fig). Indeed, this enhancement of *Desulfovibrionaceae* is notable (Fig 2c) as they can directly degrade small organic acids [90,91], reviewed by [92], like oxalate. Although we did not isolate specific strains, the maintenance of sulfate-reducing bacteria in the molly hindgut implies that oxalate-degrading capacity may be retained or enhanced in SW conditions.

The acclimation to seawater produced a temporary disturbance in homeostasis, with a notable increase in plasma oxalate concentrations followed by recovery over the 28-day period (Fig 5a); a pattern that correlates with many changes across tissues. Firstly, fish acclimated to seawater showed a significant increase in hepatic oxalate content compared to those in freshwater (Fig 5e), suggesting enhanced endogenous oxalate synthesis. Osmotic stress and/or associated metabolic rate changes at high salinity (e.g., rainbow trout and chinook salmon [93]; sockeye salmon [94]), may have led to greater oxalate generation in the liver, possibly through increased amino acid breakdown [53,95] to support increased metabolism [96]. Indeed, enhanced liver LDH activity, the main enzyme that converts glyoxylate into oxalate during amino acid metabolism in the liver [4,53], was seen after seawater acclimation (rainbow trout: [54] and tilapia: [97]).

It is important to note that oxalate measurements in intestinal and kidney samples represent composites of multiple compartments. In the intestine, measured oxalate includes contributions from intestinal tissue, luminal contents, and precipitated forms such as calcium/magnesium oxalate and bicarbonate/oxalate complexes. Changes in measured intestinal oxalate therefore likely reflect a combination of processes, including epithelial transport, precipitation dynamics, and microbial degradation. In particular, degradation of oxalate by the intestinal microbiome would be expected to lower luminal oxalate concentrations, thereby reducing the amount available for excretion and influencing systemic oxalate balance. Physically separating luminal contents from intestinal tissue (e.g., via intestinal lumen washes) would help resolve the relative contributions of epithelial transport, microbial metabolism, and oxalate precipitation but this approach is not feasible in small-bodied teleosts such as *P. latipinna* because the intestinal lumen contains very little fluid in both freshwater and seawater conditions (especially in freshwater environments due to low drinking rates). Additionally, compartmental integrity is rapidly lost following euthanasia, making separation of luminal contents from intestinal tissue impossible.

Similarly, the small size and structural simplicity of the kidney in *P. latipinna* preclude meaningful compartmental resolution of oxalate pools, necessitating the use of whole-organ homogenates. In the kidney, homogenates contain renal tissue, blood, and filtrate, making it challenging to attribute changes in measured oxalate to a specific compartment. This complexity underscores the importance of careful interpretation of oxalate data.

The plasma increase might also reflect a bottleneck in oxalate excretion. In seawater, the fish kidney drastically reduces urine volume to conserve water [10,11], with low glomerular filtration rates and the production of concentrated urine mainly for divalent ion elimination (Mg^2+^, SO_4_^2−^; [98,99,100,101,102]. This reduction in urine output means that soluble wastes have less avenue for elimination via the kidney; ultimately constraining renal excretion of oxalate as indicated by the accumulation of the metabolite in the tissue (Fig 5d) despite increased SLC26A6 expression levels for oxalate secretion (Fig 6c; also previously seen in *Takifugu obscurus* [103]). Notably, enhanced precipitation of oxalate was observed (Fig 5d), suggesting a potential increase in pH despite the general trend toward acidic urine in marine fish (typically ~1.0–2.0 pH units lower than the blood-plasma [98,104,105]). The increased renal expression of SLC26A6, which is also a Cl ⁻ /HCO₃ ⁻ exchanger could potentially enhance bicarbonate secretion into the renal filtrate, which may contribute to a more alkaline renal environment which favors oxalate precipitation. This may reflect a species-specific or context-specific adaptation for oxalate homeostasis, perhaps induced by environmental or dietary factors.

Furthermore, urinary oxalate excretion was influenced by salinity acclimation, with short-term seawater exposure increasing total and free oxalate, while long-term acclimation stabilized these levels and precipitated oxalate remained unchanged (Fig 5f), likely reflecting a shift in intestinal and renal handling. Kidney oxalate concentrations (Fig 5d) and urinary oxalate composition (Fig 5f) demonstrate coordinated but contrasting responses to salinity: prolonged seawater exposure increased renal oxalate accumulation (Fig 5d), while the urine facilitated the excretion of predominantly free oxalate (Fig 5f). This pattern suggests that the oxalate retained within the kidney is not sequestered as insoluble calcium oxalate but is instead transported into urine in a chemically stabilized, soluble state (sodium oxalate, potassium oxalate, magnesium oxalate), likely to prevent pathological crystallization and preserve renal integrity (Fig 5f). However, the authors note that given the small urine volumes obtainable from *P. latipinna*, urinary oxalate measurements should be interpreted as relative indicators of excretory trends rather than precise estimates of absolute flux. Regardless, SLC26A3 expression was not detected in the kidney fitting with previous research indicating its presence almost exclusively in the gastrointestinal tract [16].

Importantly, our findings are consistent with the hypothesis that *P. latipinna* compensates for enhanced oxalate plasma concentrations by modulating intestinal oxalate transport in response to salinity. However, these conclusions are based on changes in SLC26A3 and SLC26A6 transcript abundance and therefore reflect regulatory potential rather than direct measurements of transporter protein abundance or oxalate flux across the epithelium. This distinction is important because changes in mRNA levels do not necessarily translate to changes in protein abundance or activity, meaning that the actual capacity for oxalate transport across the intestinal epithelium may differ from what transcript measurements suggest.

Specifically, in SW-acclimated fish, transcript levels of SLC26A3 (mammalian homologue which mediates oxalate absorption [17]) was decreased in both the anterior (Fig 6a) and posterior intestines (Fig 6b) while SLC26A6 in both the anterior (Fig 6a) and posterior intestine (Fig 6b) were elevated during acclimation; although the posterior intestine eventually decreased SLC26A6 expression after 28 days when compared to the SW14 condition (Fig 6b).

Given the dual affinity of SLC26A6 exchangers for bicarbonate and oxalate anions [14], regulation of these transporters in *P. latipinna* likely modulated oxalate translocation to and from the intestinal lumen alongside bicarbonate used for osmoregulation. Indeed, it is this context that previous work has noted similar increased SLC26A6 expression (e.g., naked carp (*Gymnocypris przewalskii*) [106]; sea bream (*Sparus aurata*) [27]) and downregulation of intestinal SLC26A3 although this regulation is more variable across species (tilapia [107], sea bream [26,27], red drum [108]). While we did not directly measure oxalate flux across the intestinal epithelium in vivo, the regulation of the transporters corresponded to a predicted resulting increase in intestinal oxalate content in SW fish (relative to FW; Figs 5b & 5c) and implies that net oxalate secretion into the gut may have been higher in seawater. This was not seen by Whittamore (2020) in vitro but the lack of hormonal, nervous, and other physiological inputs may be responsible for this contradiction and reveals the potential importance that in vivo conditions might be important for this process. It is important to note that the diet may be an additional source of oxalate and while fish across all conditions were fed the same type and amount of food, the digestive efficiency of fish acclimated to SW has been shown to increase compared to FW [109,50]. Thus, the higher intestinal oxalate concentrations observed in seawater compared to those in freshwater (Figs 5b & 5c) might also be attributed to higher digestive extraction not just enhanced oxalate secretion into the lumen. This requires future studies to examine the potential confounding role this plays in intestinal oxalate concentrations.

Functional characterization of teleost SLC26A6 paralogs has provided direct evidence that these exchangers mediate oxalate transport [103]. Kato and colleagues cloned mfSlc26a6A, mfSlc26a6B, and mfSlc26a6C from *Takifugu obscurus* and expressed them in *Xenopus laevis* oocytes, where they facilitated electrogenic Cl ⁻ /oxalate² ⁻ exchange [103]. Oxalate-induced membrane currents were observed in oocytes expressing these paralogs but not in water-injected controls, and current–voltage relationships were consistent with an active anion exchange mechanism, with reversal potentials near the equilibrium potential for chloride [103]. Furthermore, it was shown that teleost SLC26A6 paralogs are capable of mediating Cl ⁻ /oxalate² ⁻ exchange in a heterologous expression system and localized Slc26a6A to the apical membrane of renal tubules [103], consistent with a role in epithelial secretion. Their findings demonstrate that teleost SLC26A6 paralogs directly mediate oxalate transport, in addition to their roles in Cl ⁻ /SO₄²⁻ and Cl ⁻ /HCO₃ ⁻ exchange, underscoring their potential contribution to oxalate regulation in osmoregulatory tissues [103]. Although teleost SLC26A3 orthologues are expressed in intestinal epithelium, research has largely focused on mammals, and their role in oxalate transport in teleosts has yet to be established. As the primer sets for SLC26A3 and SLC26A6 targeted regions conserved across all isoforms, the qPCR results reflect the total activity of all isoforms for each gene, and it was not possible to resolve the activity of individual isoforms in the current study.

Broad-spectrum antibiotic treatment provided a mechanistic test of our hypothesis that gut bacteria are essential for maintaining oxalate homeostasis in *P. latipinna*. The absence of a detectable 16S rRNA PCR product in antibiotic-treated fish was used as a qualitative confirmation that antibiotic exposure altered intestinal bacterial DNA. This approach was intended to verify that the treatment had a reasonable effect on the gut microbiota, rather than to provide a quantitative measure of bacterial load or the extent of microbial depletion. It is important to note that antibiotic exposure in this study reflects microbiome disruption rather than complete microbial elimination, as microbial load was not directly quantified and residual bacteria, resistant taxa, or microbial enzymes may have persisted and contributed to the observed patterns. This pattern is consistent with altered oxalate handling following microbiome disruption rather than complete loss of microbial contributions.

Disruption of the microbiota resulted in elevated intestinal, plasma, and renal oxalate levels (Figs 7a–7d), and altered transporter expression (Fig 8) which support the interpretation that bacteria are not merely correlated with oxalate handling, but may play a contributory role in oxalate degradation and clearance. Specifically, there was a ~ 15-fold upregulation of SLC26A6 in the kidney of seawater fish treated with antibiotics (Fig 8c), accompanied by elevated renal oxalate levels (Fig 7d). These results suggest a possible compensatory renal clearance mechanism, whereby, in the absence of microbial degradation, oxalate is retained systemically and redirected to the kidney — a strategy that might be physiologically costly (e.g., elevated plasma oxalate concentrations; Fig 7a).

Disruption of the gut microbiome with antibiotics slightly increased total urinary oxalate, while free and precipitated oxalate concentrations remained unchanged, consistent with the idea that urinary oxalate excretion in seawater is already constrained [2,10,11], and that renal clearance rather than microbial degradation limits urinary oxalate output under these conditions (Fig 7f). Together, these results are consistent with seawater acclimation producing a dual response of renal oxalate accumulation and enhanced soluble urinary excretion, representing a coordinated strategy to manage elevated systemic oxalate loads.

Intestinal SLC26A6 expression did not increase (Figs 8a & 8b) despite higher plasma oxalate levels, contradicting the expectation of a compensatory upregulation. This unexpected result suggests that antibiotics may directly impair SLC26A6 expression in the gut, highlighting a complex host-microbe interaction. However, the downregulation of SLC26A3 (Figs 8a & 8b) may represent a host attempt to limit oxalate absorption under conditions of microbial loss. In parallel, we observed a notable increase in hepatic oxalate concentrations (Fig 7e), suggesting that antibiotic treatment may have altered host metabolism (particularly amino acid metabolism and LDH activity) further augmenting oxalate burdens beyond the loss of intestinal clearance. This aligns with previous findings in zebrafish, where antibiotic exposure ((SMX (260 ng/L) and OTC (420 ng/L) over a six-week period) increased metabolic rate [110] and enhanced amino acid catabolism in the liver may increase endogenous oxalate production. Altogether, these findings suggest that the loss of microbial oxalate degradation cannot be fully compensated by host transport mechanisms. These findings closely parallel the situation in mammals with enteric hyperoxaluria: humans receiving broad-spectrum antibiotics can lose *Oxalobacter formigenes* and other oxalate degraders, resulting in elevated urinary oxalate and a risk of kidney stones [38]. Additionally, the observed increase in free oxalate levels in the posterior compared to the anterior intestine in fish from the SW14 + Antibiotics condition (S2 Fig), contrasting with the pattern seen in SW14 fish with an intact microbiome (Fig 5 vs. Fig 7), suggests a potential role for the posterior intestinal microbiome in oxalate handling.

Ecologically, these findings underscore the potential vulnerability of wild fish to antibiotic pollution or other dysbiosis-inducing stressors. A transient loss of microbial oxalate degraders could trigger oxalate imbalance, stressing renal pathways, and compromising overall osmoregulatory efficiency. In this context, the dysbiosis acts as a metabolic lesion, revealing a potentially non-redundant, essential role of gut microbes in detoxifying dietary and endogenous oxalate.

## Conclusions

This study presents the first comprehensive analysis of oxalate metabolism in a euryhaline teleost, *Poecilia latipinna*, highlighting a coordinated host-microbe system that appears to mitigate oxalate accumulation across salinities. We show that oxalate is managed via (1) salinity-dependent intestinal transport—likely through SLC26-family exchangers promoting secretion in seawater—and (2) a regionally specialized gut microbiome putatively involved in oxalate degradation in the posterior intestine. In seawater, the gut may function as an auxiliary excretory organ, akin to the mammalian colon, using shared transporters to balance ion and oxalate flux. When intact, this system buffers against salinity-driven disruptions; however, antibiotic treatment disrupts microbial communities and is consistent with impaired microbial oxalate degradation (as inferred from altered oxalate levels and transporter expression, rather than direct sequencing data), resulting in systemic oxalate stress and underscoring microbial importance in metabolic homeostasis.

These findings also reveal a novel intersection between osmoregulation and waste management in fish. By co-regulating transporters for both salt and oxalate and shifting excretion from kidney to gut, *P. latipinna* integrates metabolic and osmoregulatory demands. Shifts in oxalate-degrading bacterial families across salinities suggest functional redundancy and resilience in the gut microbiome. Furthermore, we provide the first evidence that osmoregulatory demands shape gut microbial composition and are associated with enhanced predicted oxalate-catabolizing capacity in the posterior intestine.

This work advances our understanding of teleost physiology and the ecological relevance of host-microbe interactions. Future directions include isolating oxalate-degrading bacteria, probing SLC26 regulation, and assessing dietary impacts. Applied implications include probiotic strategies to prevent oxalate-related disorders in aquaculture. Together, these results support a coordinated host–microbiome response to salinity that reshapes oxalate handling capacity, while recognizing that functional fluxes and microbial metabolism remain to be tested directly in future studies. Overall, oxalate metabolism emerges as a key component of environmental adaptation and symbiosis in teleosts.

## Supporting information

S1 Table Experimental design detailing treatment groups, tank allocation, total number of fish per treatment, and the number of fish analyzed for each endpoint. (TIF)

S2 Table Primer sequences, annealing temperatures, and hypervariable region (universal bacterial primers) for all the primer sets used in this study. (TIF)

S1 Fig Mapping of primers and conserved regions across SLC26A3 and SLC26A6 isoforms in *Poecilia latipinna.* SLC26A3 isoforms (A) and SLC26A6 isoforms (B) are represented as grey tracks with nucleotide positions along the x-axis. Conserved regions identified from sequence alignment are shown as shaded blue rectangles. Forward primers are indicated in green and reverse primers in red at their respective binding sites.
(TIF)

S2 Fig Intestinal zonation patterns of the total (black), free (dark grey), and precipitated (light grey) oxalate concentrations (µmol mg wet tissue weight-1) in the anterior and posterior intestines of the FW14 (a), SW14 (b), SW28 (c), and SW14 + Antibiotics (d) conditions. The bars represent column means ± SEM (n-values). Within each panel, bars that share letters are not statistically different. For all the panels, the datasets were square root-transformed to pass normality and mixed-effects linear regression models were employed. In all analyses, individual fish were treated as the unit of observation, and tank identity and sex were included in the models to account for potential clustering; no significant tank or sex effects were detected, so fish were treated as independent observations for inference.
(TIF)

S3 Fig Intestinal zonation patterns of SLC26A3 (striped bars) and SLC26A6 (cross-hatched bars) gene expression levels across the FW14 (a), SW14 (b), SW28 (c), and SW14 + Antibiotics (d) conditions. The relative gene expression levels are expressed as percentages with the anterior intestine in each condition set to 100%. The bars represent column means ± SEM (n-values). Within each panel, bars that share letters are not statistically different. For SLC26A3, all four panels did not pass normality and generalized additive models were used. For SLC26A6, the datasets for all four panels were log(10)-transformed to pass normality and mixed-effects linear regression models were utilized. In all analyses, individual fish were treated as the unit of observation, and tank identity and sex were included in the models to account for potential clustering; no significant tank or sex effects were detected, so fish were treated as independent observations for inference.
(TIF)

S4 Fig Analyses of the gut microbiome of sailfin mollies acclimated to either freshwater or seawater for 14 days. The relative abundance (%) of bacterial families that are significantly different (ANCOM-Level 5) in the anterior intestines (a) and posterior intestines (b) across the freshwater and seawater conditions. The relative abundance (%) of bacterial families that are significantly different (ANCOM-Level 5) between the anterior and posterior intestine of sailfin mollies acclimated to either the freshwater (c) or seawater (d) condition is also shown.
(TIF)

S5 Fig Predicted differential KEGG pathways across environmental salinities and intestinal sections. All predicted differential KEGG pathways (Level 2) under the metabolism category between the freshwater and seawater environments for the anterior (a) and posterior (b) intestines; as well as intestinal zonation between the anterior and posterior intestines in freshwater (c) and seawater (d). The top 15 (based on highest mean proportions) predicted differential KEGG pathways (Level 3) under the metabolism category between the freshwater and seawater environments for the anterior (e) and posterior (f) intestines; as well as intestinal zonation between the anterior and posterior intestines in the freshwater (g) and seawater conditions (h). The bar plot on the left depicts each KEGG pathway’s mean proportion (%) and on the right, the difference in mean proportion (%) between the two groups, the 95% confidence interval, and p-value are shown.
(TIF)
